# Characterizing PET CT patterns and bacterial dissemination features of tuberculosis relapse in the macaque model

**DOI:** 10.1128/iai.00177-25

**Published:** 2025-06-23

**Authors:** Pauline Maiello, Collin Diedrich, Tara Rutledge, Mark Rodgers, Kara Kracinovsky, H. Jacob Borish, Alexander White, Forrest Hopkins, Michael C. Chao, Edwin Klein, Sarah Fortune, JoAnne L. Flynn, Philana Ling Lin

**Affiliations:** 1 Department of Microbiology and Molecular Genetics, University of Pittsburgh School of Medicine 541988, Pittsburgh, Pennsylvania, USA; 2 Center for Vaccine Research, University of Pittsburgh School of Medicine 588296, Pittsburgh, Pennsylvania, USA; 3 Department of Pediatrics, Children’s Hospital of Pittsburgh of the University of Pittsburgh Medical Center, University of Pittsburgh School of Medicine 209879, Pittsburgh, Pennsylvania, USA; 4 Department of Immunology and Infectious Diseases, Harvard T. H. Chan School of Public Health 1857 , Boston, Massachusetts, USA; 5 Division of Laboratory Animal Research, University of Pittsburgh School of Medicine 12317 , Pittsburgh, Pennsylvania, USA; 6 Broad Institute, Harvard University & Massachusetts Institute of Technology 2167, Cambridge, Massachusetts, USA; 7 Ragon Institute of MGH, MIT, and Harvard 200750 , Cambridge, Massachusetts, USA; Rutgers-New Jersey Medical School, Newark, New Jersey, USA

**Keywords:** tuberculosis, relapse, PET CT, HIV

## Abstract

Tuberculosis (TB) relapse after appropriate drug treatment is poorly understood but critical to developing shorter treatment regimens. Using a cynomolgus macaque model of human TB, macaques with active TB disease were treated with a short course of isoniazid and rifampin and subsequently infected with SIV. Serial clinical, microbiologic, immunologic, and position emission and computed tomography (PET CT) assessments were performed to identify risk factors of relapse. Of the 12 animals, eight developed radiologically defined relapse, including four that had clinical and/or microbiologic signs. Greater gross pathology and bacterial burden were observed in relapse animals. PET CT characteristics before, during, and at the end of the treatment were similar among relapse and non-relapse animals. We show that complete sterilization or very low Mtb burden is protective against SIV-induced TB relapse but cannot be predicted by PET CT. Using barcoded *M. tuberculosis*, we found that Mtb dissemination during relapse originated from both lung and thoracic lymph nodes, underscoring the importance of lymph nodes as a reservoir. By matching barcoded Mtb and serial PET CT, we also demonstrate that not every site of persistent Mtb growth after drug treatment is capable of dissemination and relapse, underscoring the complex nature of drug treatment and relapse.

## INTRODUCTION

In 2023, ~8.2 million people were newly diagnosed with symptomatic, active tuberculosis (TB) caused by *Mycobacterium tuberculosis* (Mtb), and 1.2 million deaths occurred (1). Standard TB treatment involves 6 months of multiple drugs and has a cure rate of 85% (1). However, an estimated 7% of all patients develop TB relapse (2), although relapse from primary infection after treatment can be difficult to distinguish from reinfection without molecular testing of serial samples (3). In a meta-analysis from 1980 to 2020 addressing reinfection/relapse within 2 years, 70% of the cases were from relapse and 30% from reinfection (4). Using a multi-predictor analysis from a large pool of published patients, HIV infection (OR: 2.6, 95% CI: 1.4–4.6), baseline cavitary disease with a positive smear at 2 months of treatment (OR: 2.3, 95% CI: 1.3–4.3), and baseline cavitary disease with a positive culture at 2 months post-treatment (OR: 2.1, 95% CI: 1.2–3.8) had the highest odds of relapse, but these factors only account for 10% of all relapse cases (2). TB relapse is clearly underappreciated and not well understood.

Given that the majority of relapse cases have no identified risk factor, better models and tools are needed to understand and prevent relapse (reviewed in references 5–7). Positron emission tomography (PET) using ^18^F-fluorodeoxyglucose (FDG) as a metabolic PET probe with computed tomography (CT) has been used to follow TB disease progression/treatment with promising results in predicting treatment success (8–11). This is particularly important as the development of shorter treatment regimens is a key TB eradication strategy, but long-term patient follow-up is difficult and expensive. Models that can predict TB cure and improve our basic understanding of the events that control and prevent relapse are direly needed.

Serial FDG-PET CT in the macaque model of TB can be extremely useful in understanding human Mtb infection, HIV-TB coinfection, response to drugs, and vaccine efficacy (12). Recognizing that HIV is a major risk factor for relapse, we modified our TB-SIV coinfection animal model with the aim of examining TB relapse. Cynomolgus macaques infected with low-dose Mtb develop the full spectrum of outcomes and pathology seen in humans (13–15). Macaques with clinically defined active TB were treated with isoniazid (INH) and rifampin (RIF) for 2 months, followed by a 1-month washout period and then SIV infection, and serial assessments were performed. Eight of the 12 animals developed radiographically defined relapse resulting in greater gross pathology disease and bacterial burden. Using barcoded Mtb and PET CT, we provide insights into the dynamic and complex nature of bacterial dissemination during relapse.

## MATERIALS AND METHODS

### Animals and study design

**Fig 1:**
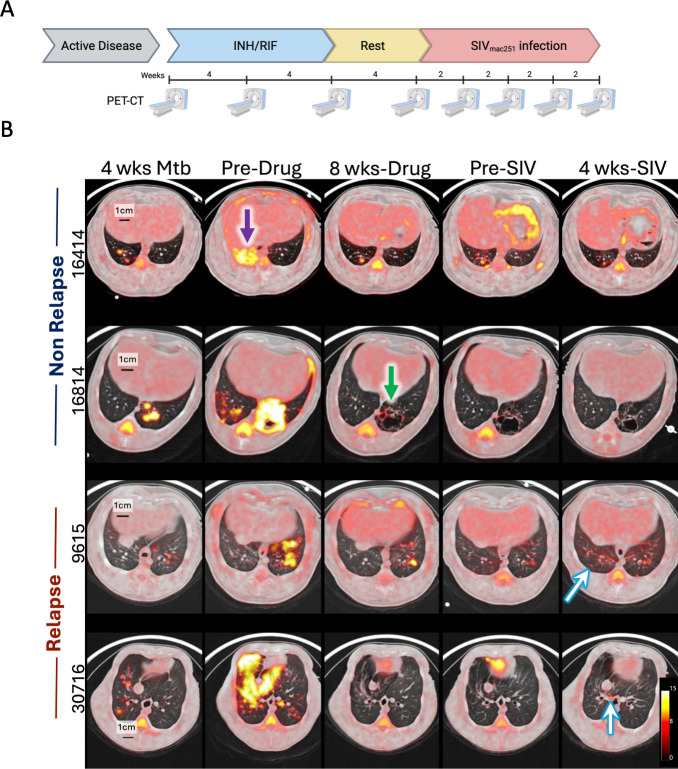
Experimental design and serial PET CT images from animals with active TB before and during treatment and relapse. (**A**) Experimental study design. Animals with active TB disease were given a 2-month course of isoniazid (INH) and rifampin (RIF), followed by 1 month of no drugs, after which SIV infection occurred. Serial PET CT was performed prior to, during drug treatment, before, and after SIV infection as shown above. Created with Biorender.com. (**B**) Serial PET CT images of TB disease in cynomolgus macaques at 4 weeks post-Mtb infection, pre-drug treatment, 8 weeks post-drug treatment, pre-SIV infection, and 4 weeks post-SIV infection. Axial images of the chest demonstrate the degree of TB-associated lung involvement and FDG activity (shown in yellow) among two animals without evidence of relapse (16414 and 16814) and animals with relapse (9615 and 30716). Purple arrow shows a consolidation, and green arrow shows a cavity. Blue and white arrows point to granulomas that appeared on the scan after SIV infection. SUV (standardized uptake value) scale bar is shown in the bottom right.

Adult Chinese cynomolgus macaques (*Macacca fasicularis*) (Valley Biosystems, Sacramento, CA) were screened for other infectious co-morbidities (e.g., SIV, SRV, parasites, and Mtb) before infection. Animals were infected with molecularly barcoded (~15 CFU) Mtb Erdman strain via bronchoscopic instillation that was confirmed by a PET CT scan (16). Animals with clinically defined active TB disease (13, 15) (i.e., having signs of disease [cough, weight loss, anorexia], evidence of Mtb growth by gastric aspirate [GA] or bronchoalveolar lavage [BAL], and progressive disease by PET CT imaging) were treated with human equivalent doses of isoniazid (INH, 15 mg/kg/dose daily by mouth) and rifampin (RIF, 20 mg/kg/dose daily by mouth) for 8 weeks. All animals received at least 85%–100% of their doses. After a 1-month drug-free period, animals underwent SIV_mac251_ infection (swarm) (1.67 × 10^5^ viral RNA copies, intravenous) for 8 weeks until necropsy (Fig. 1A). (Table S1) Serial clinical, immunologic, microbiologic, and radiographic assessments were performed for the entire duration of the study. Blood was obtained via venipuncture for isolation of peripheral blood mononuclear cells (PBMC) every 1–4 weeks and bronchoalveolar lavage (BAL) monthly for flow cytometric analysis as previously described (17).

### PET CT acquisition and analysis

*In vivo* progression of disease was monitored and quantified using the microPET Focus 220 preclinical PET scanner (Siemens Medical Solutions) and a clinical 8 slice helical CT scanner (NeuroLogica Corp) with previously published methods and metrics (18). Scans were performed before treatment and every 2–4 weeks thereafter. (No scans were performed prior to Mtb infection due to budgetary constraints.) PET CT analysts (P.M., A.G.W., and H.J.B.) were blinded to the treatment group. Relapse was defined as the development of new lung lesions identified by PET CT (and confirmed at necropsy) after completing INH/RIF treatment.

### Quantification of disease, Mtb burden, SIV RNA, and Mtb genome isolation with barcode mapping

Gross pathology of TB disease was quantified at necropsy using a previously validated method (19). Scan-matched lung granulomas and other pathologies from other sites (e.g., lymph nodes, liver) were harvested. Tissues were homogenized into single-cell suspension for immunologic assays and bacterial burden (19). Mtb burden was quantified in colony-forming units (CFU) from individual sites (19). Lymph node burden (“LN CFU”) is the sum of CFU from all thoracic lymph nodes. Extrapulmonary score (EP score) is a quantitative estimate of extrapulmonary involvement (e.g., liver, paracostal abscess, and kidney) based on bacterial growth and gross or microscopic evidence of TB (19). Total bacterial burden includes the sum of CFU from the lymph nodes (thoracic and extrapulmonary) and thoracic cavity (grossly normal lung, granulomas, involved lung, or diaphragm granulomas). Bacterial burden for animal 16714 was omitted due to contamination. Samples with Mtb growth were sequenced for barcode identification using prior methods (16). Each barcode from disease sites was mapped three-dimensionally (estimated Cartesian coordinates by PET CT) using Osirix imaging software (OsiriX MD, version 12.0.3). New lesions identified after SIV infection were called “relapsed lesions” and assumed to be disseminated from pre-existing sites of disease. CD4 counts and SIV viral RNA copies from plasma and tissues were also measured as previously published (17).

To determine whether the severity and distribution of disease involvement prior to treatment were associated with relapse, spider plots of each animal were generated by connecting the locus of points created by plotting a set of PET CT-based disease measurements on a polar axis (Python 2.7.15, Matplotlib 2.2.3). The angular coordinates correspond to the metric, and the radial coordinate gives the value of the metric normalized to the maximum value of that metric in the data set. The maximum value of each metric was determined across all animals and time points. The value of the radial coordinate is the value of the metric measured in that specific animal at the specific time point divided by the maximum value of that metric across all animals and time points. The measurements included the following: total lung FDG activity (square-root-transformed, 0–695.312908), number of extrapulmonary sites (0–5), lung lobes involved (1–7), necrotic thoracic lymph nodes (0–6), FDG-active thoracic lymph nodes (0–13), lung cavity (0 = no, 1 = yes), granuloma clusters (0 = no, 1 = yes), consolidation (0 = no, 1 = yes), and bilateral lung disease (0 = unilateral, 1 = bilateral).

### Flow cytometry analysis

Flow cytometry was performed on PBMC, BAL, and tissues using a combination of antibodies (Table S2A), as described (17). PBMC and BAL cells were stimulated with Mtb ESAT6-CFP10 overlapping peptide pools (10 µg/mL of each peptide) and/or only media (RPMI + 10% hAB) prior to staining. Data acquisition using an LSR II (BD) was performed, and FlowJo Software v.9.7 (Treestar Inc, Ashland, OR) was used for analysis. The gating strategy for cytokines and cytotoxic markers is shown in Fig. S1.

### Circos plots

For each animal, the identified barcodes in each tissue and associated metadata including tissue anatomical location and time of lesion detection (pre- and post-SIV infection) were converted into text formats using custom scripts compatible with plotting by Circos software (20). Plots were manually adjusted such that links between tissues sharing barcodes were drawn, when possible, to originate from lung sites detected early by PET CT with the highest CFU burden; however, other lung sites with the same barcode may also be the source of dissemination between tissues. Custom scripts and relevant configuration files used to generate the Circos plots are available upon request.

### Statistical analysis

Shapiro-Wilk test was used to test for normality. Mann-Whitney test was used for non-paired data, and Wilcoxon matched pairs-signed rank test was used for any paired data for comparing two groups. For longitudinal PBMC and BAL data, groups were compared at each time point with Mann-Whitney tests (not adjusted for multiple comparisons). Longitudinal PET data were analyzed using a mixed-effects model fit using Restricted Maximum Likelihood (REML) with Dunnett’s multiple comparisons test adjusted *P*-values reported. Categorical data were analyzed using Fisher’s exact test. For counts (including cell counts, CFU, and FDG activity), the data were first transformed (adding one to the entire data set) so that zeroes could be visualized and analyzed on a log_10_ scale. All statistical tests are two-sided, and significance was established at *P* ≤ 0.05. Figures, graphs, and statistical tests were performed in GraphPad Prism Mac OSX (Version 10.1.1, GraphPad San Diego, CA) or JMP Pro 17.2.0.

## RESULTS

### PET CT-defined relapse is associated with a higher bacterial burden

Macaques with clinically defined active TB disease have a range of radiographic patterns that are seen in human TB, including cavities (e.g., 16814, Fig. 1B, green arrow), granulomas of various sizes, multiple lung lobe involvement, lung consolidation (e.g., 16414 pre-drug image, Fig. 1B, purple arrow), tree-n-bud pattern, lymphadenopathy with or without necrosis, etc. The standard treatment of drug-susceptible TB includes 2 months of rifampin (RIF), isoniazid (INH), pyrazinamide, and ethambutol, followed by 4 more months of RIF and INH. As this was a pilot study for model development with a limited number of animals (*n* = 12), a short course of treatment with only RIF and INH (the two backbones of standard TB treatment) for 2 months was chosen to prevent complete sterilization of Mtb and increase the likelihood of relapse. Following treatment, macaques had a 1-month, drug-free period and then SIV infection (Fig. 1A). During drug treatment, there was an overall reduction in lung inflammation (measured as total lung FDG activity), but no difference in change of inflammation over time between animals that would later develop relapse compared with non-relapse animals (Fig. S2A). Although inflammation of individual lymph nodes varied in animals over time, on average, thoracic lymph node inflammation (measured as standard uptake volume ratio, SUVR) decreased during treatment (fold change mean: no-relapse 0.95, relapse 0.61) (Fig. S2B). Lesion-specific reductions in size and inflammation were also observed, especially in large consolidations and cavities (Fig. 1B). Only one animal (31216, relapsed) had a persistent consolidation but with reduced size and metabolic activity during drug treatment, while the remaining animals had near resolution of consolidations on the scan. Lesional changes associated with treatment varied over time (i.e., some lesions took longer to improve than others) and were independent of bacterial burden at necropsy (Fig. S2C) as expected given the heterogeneity in bacterial burden and host response in individual granulomas (21–23).

**Fig 2:**
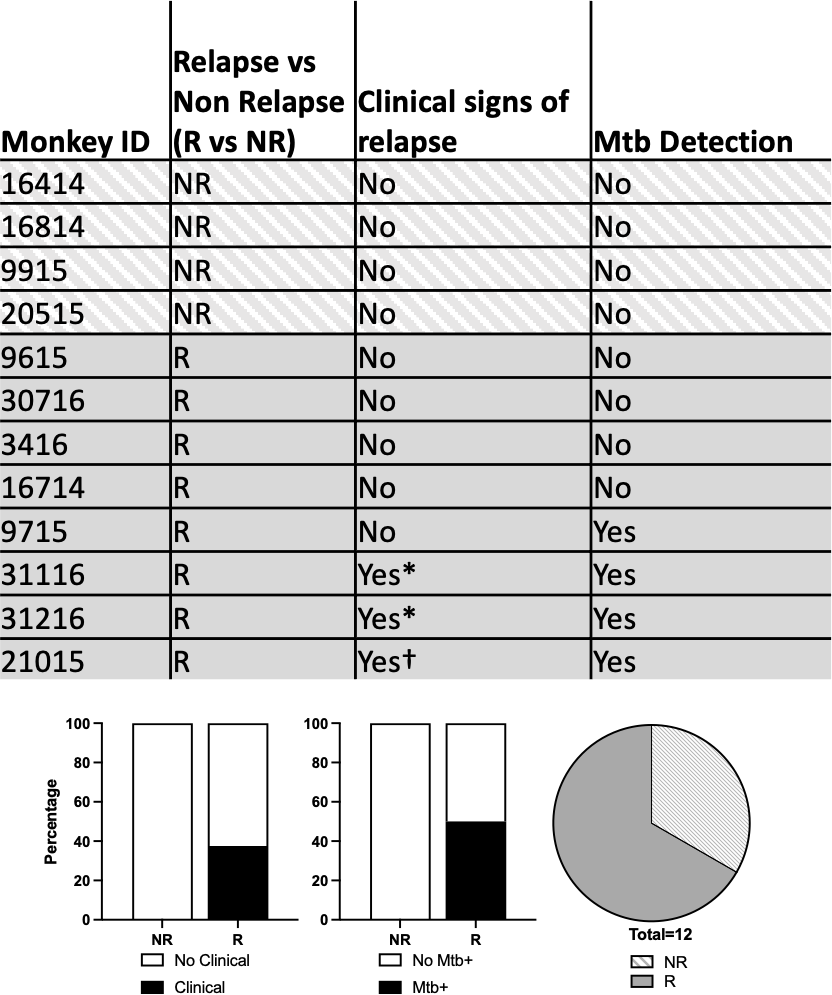
Clinical and microbiological evidence of relapse after SIV infection among radiologically defined relapse and non-relapse animals. Mtb detection refers to microbiologic evidence (Mtb detected) in either gastric aspirate (GA) or bronchoalveolar lavage (BAL) samples. *Increased respiratory effort. †Irregular respirations. Bar graphs represent the percentage of animals in each outcome group with clinical or microbiologic evidence of relapse post-SIV infection.

**Fig 3:**
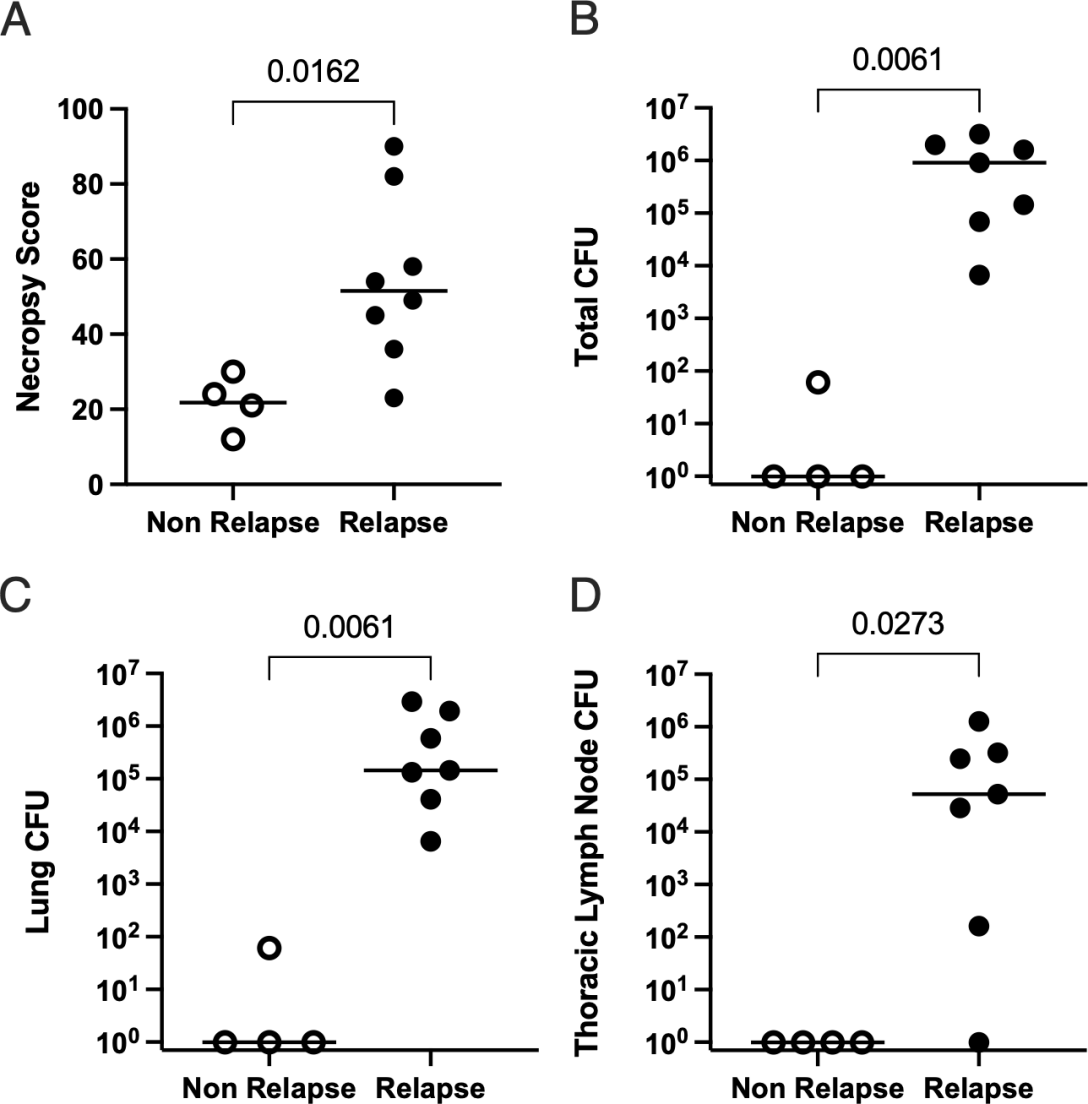
PET CT-defined relapse is associated with greater gross pathology at necropsy (measured by necropsy score) (**A**), total Mtb burden (measured as CFU, colony forming units of Mtb) (**B**), lung Mtb burden (**C**), and thoracic lymph node burden (**D**) compared with non-relapse animals. *P*-values shown were determined by the Mann-Whitney test. Each dot represents an animal, and the lines shown are medians.

Relapse TB was defined as the development of a new lung granuloma detected by PET CT (and confirmed at necropsy) after SIV infection (Fig. 1B; Fig. S3A). Eight of the 12 animals developed relapse. Four of the eight relapse animals had microbiologic and/or clinical evidence of disease (i.e., tachypnea and Mtb detected); non-relapse animals had no such signs (Fig. 2). At necropsy, relapse macaques had greater pathology and more Mtb CFU (95% CI of log_10_ difference between medians 3.8 and 6.3) than non-relapse animals, particularly in the lung and thoracic lymph nodes (Fig. 3; Fig. S3B through D). Serial PET CT images allowed us to track granulomas that appeared before and after treatment. Relapse animals had a significantly higher frequency of granulomas (median of 68%) with viable Mtb at necropsy. Among the new granulomas that appeared during relapse, 84% had viable Mtb growth. In contrast, only one of the four non-relapsed animals had Mtb growth at necropsy in a single granuloma (184 CFU) (Fig. S3E and F). Thus, although killing all viable Mtb in every anatomic reservoir would be an optimal strategy for drug treatment, some viable Mtb are likely to persist after “successful” treatment, emphasizing the importance of ongoing immune control in preventing relapse.

### Pre-treatment radiographic characteristics are not associated with relapse risk

**Fig 4:**
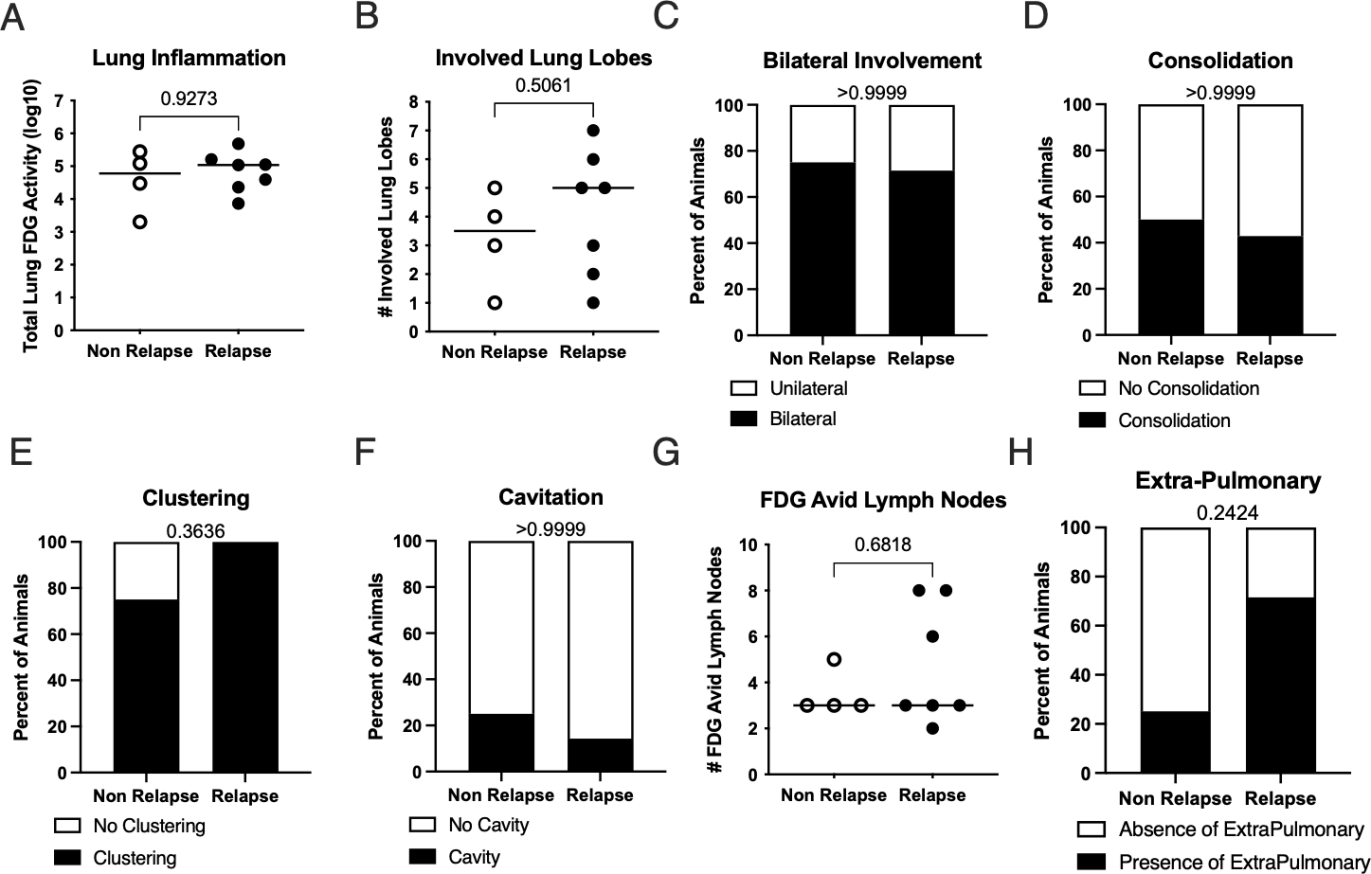
PET CT characteristics prior to TB drug treatment. PET CT characteristics were similar between non-relapse (*n* = 4) and relapse (*n* = 7; one animal could not be scanned prior to drug treatment) animals that included total lung inflammation (measured using FDG activity) (**A**), number of involved lung lobes (**B**), proportion of unilateral or bilateral lung involvement (**C**), proportion of animals with lung consolidation as a CT feature (**D**), proportion of the animals with clusters of granulomas. as a feature of TB disease on PET CT (**E**), proportion of animals with cavitary disease on PET CT scan (**F**), number of FDG-avid (as a marker of inflammation) mediastinal lymph nodes (**G**), and proportion of animals with PET CT findings of extrapulmonary disease prior to drug treatment (**H**). *P* values are based on Mann-Whitney (**A, B, G**) or Fisher’s Exact (**C through F, H**) tests.

We sought to determine whether PET CT characteristics before TB treatment were associated with relapse. We previously published that total lung inflammation (total lung FDG activity) correlates with bacterial burden (19), and this measure was similar between relapse and non-relapse animals before treatment (Fig. 4A). To determine whether the extent of lung or lymph node involvement prior to treatment was associated with relapse, we compared PET CT features of disease severity including the number of lung lobes involved, presence of bilateral lung involvement, consolidation (defined as a lesion greater than 2 cm), clusters of TB lesions, cavity, metabolically active thoracic lymph nodes, and extrapulmonary disease (Fig. 4B through H). All these parameters were similar between relapse and non-relapse groups, as were the reductions in total lung FDG and lymph node FDG (Fig. S2). To examine the combined effect of disease severity, spider plots of each feature were generated with a score based on the extent of each disease feature. Scores were similar between groups before SIV-induced relapse (Fig. S4). Not surprisingly, the severity score was higher in relapse animals at necropsy (Fig. S4D).

Given that our prior data showed that TB granulomas are heterogeneous within the same host and can independently influence disease risk (21–24), we examined individual granuloma characteristics. Maximum granuloma size and/or maximum SUV (metabolic avidity) per animal before treatment and/or after treatment was not associated with relapse (Fig. S5). We then took advantage of serial PET CT scanning data in these animals to examine whether other unique PET CT features such as the resolution of granulomas were associated with relapse risk. Interestingly, all four of the non-relapse animals had at least one lung granuloma resolve completely (defined as the absence of lesion seen by PET CT on at least two subsequent scans and not found at necropsy) on PET CT, but only one of eight relapse animals had lesion resolution (100% vs 12.5%, *P* < 0.01, Fisher’s Exact).

### Distribution of barcodes during relapse

**Fig 5:**
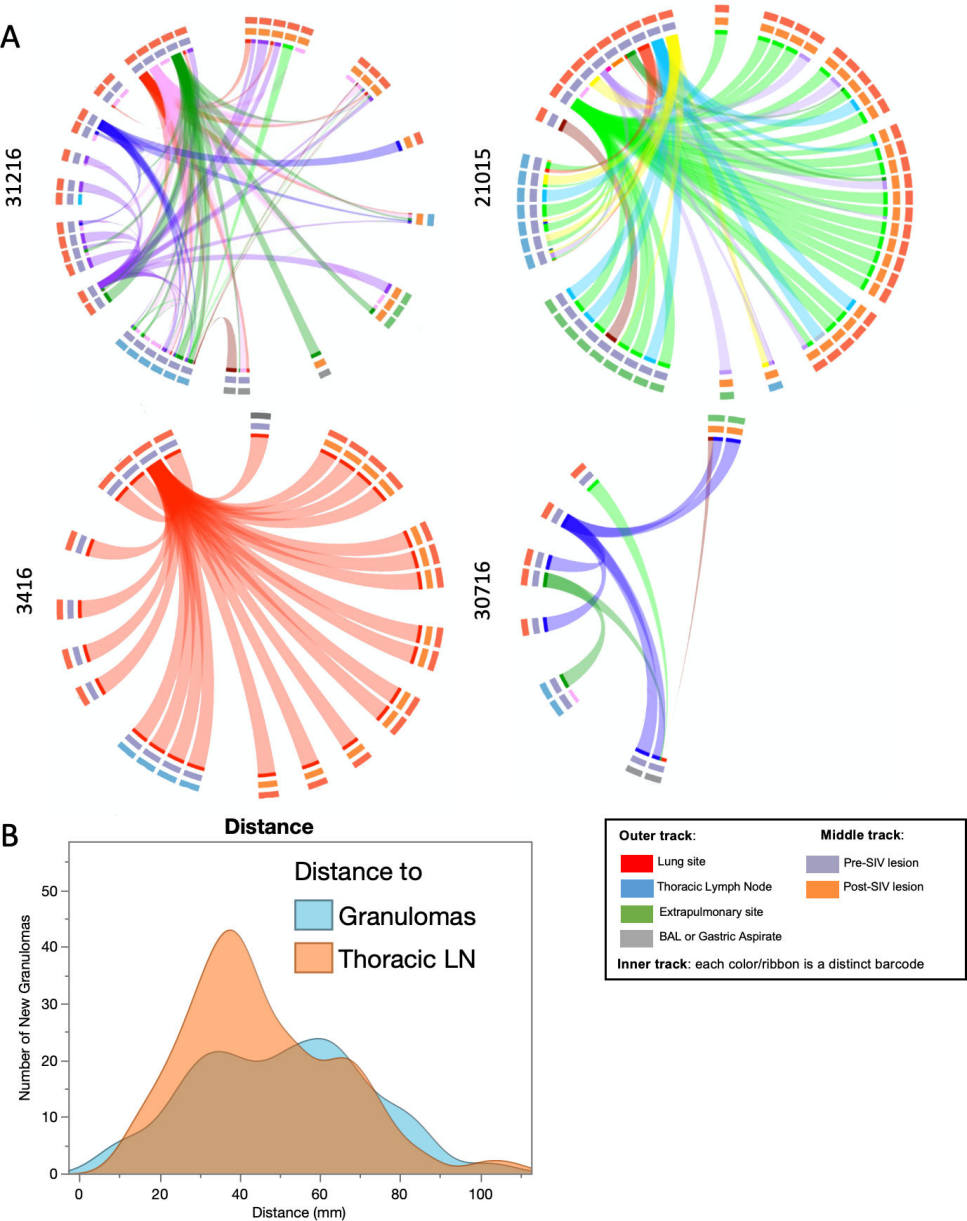
Distribution of barcodes by anatomical compartment. (**A**) Circos plots representing barcoded Mtb detected in tissues (inner track), with ribbons representing tissues containing the same barcodes. The middle track represents the timing of the sample seen on scans (pre- or post-SIV infection), and the outer track represents the anatomical compartment associated with each tissue. Distinct barcodes are represented by a different color. (**B**) New granulomas appear to be closer in distance to founding (seen at 4 weeks post-Mtb infection) lymph nodes (green) than founding granulomas (blue). Y-axis shows the number of granulomas sharing barcodes with founding granulomas and lymph nodes, and the x-axis shows the distance between the new granuloma and founding granuloma/lymph node.

We previously established that each individual granuloma is established by a single bacillus, distinguishable by a molecular barcode, whereas thoracic lymph nodes often have multiple barcodes due to migration of Mtb from lung granulomas (16, 22, 25). Accordingly, barcoded Mtb libraries allow us to track the dissemination of individual Mtb bacilli. At necropsy, we harvested PET CT scan-matched lesions from lung, thoracic lymph nodes, and other tissue sites while discriminating whether they appeared before SIV infection (or “pre-SIV”) or during relapse (or “post-SIV”). Barcodes recovered from scan-matched granulomas appearing after SIV infection were mapped to both thoracic lymph nodes and lung granulomas identified before SIV infection (Fig. 5A; Fig. S6 and S7). We found that relapse could be dominated by a single barcode strain (monkey 3416) or multiple strains. In the case of animal 31216, one of the barcodes identified from a relapsed granuloma could be matched only to a pre-treatment thoracic lymph node. These data emphasize the importance of eradicating all viable Mtb from both lung and lymph nodes. Eliminating viable Mtb within lymph nodes is particularly important because they exhibit reduced bacterial killing during drug treatment (26). However, not all barcodes recovered from pre-SIV lesions (i.e., existed during drug treatment) disseminated to relapse sites, indicating that not all viable Mtb lesions after drug treatment were capable of dissemination even during SIV infection. In fact, only 42% (median, range: 0%–100%) of barcodes from pre-treatment lung granulomas were observed in relapse granulomas, highlighting the independent risk of each granuloma to disseminate. Finally, we investigated the proximity of new granulomas to the (“pre-SIV”) originating granuloma or lymph node using barcode data and XYZ coordinates from PET CT scans before and after drug treatment (Fig. S6 and S7). New granulomas during relapse appeared to be closer in distance to thoracic lymph nodes than to old granulomas matched by barcode (Fig. 5B).

### Higher SIV RNA levels observed in relapse granulomas and lymph nodes though immune responses did not distinguish relapse outcomes

We found that plasma SIV viral loads were similar between relapse and non-relapse animals during the course of SIV infection, as were peripheral blood CD8 and CD4 T cells (Fig. S8A and B). In general, neither lung nor systemic immune responses distinguished relapse and non-relapse cases. Granuloma-specific Th1 cytokine responses among CD4 and CD8 T cells were similar between relapse and non-relapse animals (Table 1; Fig. S9). Th1 (IFN-γ, IL-2, TNF), IL-10, and IL-17 T cell responses were also similar in the airways, although animals that would later develop relapse had lower IL-10 production among alveolar macrophages before SIV infection (Table 2; Fig. S10 and S11). Blood immune responses were similar between relapse and non-relapse animals with some exceptions (Table 3; Tables S3 to S6; Fig. S13 to S20). For example, relapse animals had higher frequencies of naïve antigen-specific IFN-γ CD4 T cells but lower production of IL-17 and IL-10 producing CD8 T cells (Table S3). Lower frequencies of blood terminally differentiated (CD27−/CD45R+) CD4 and CD8 T cells were observed in the non-relapse animals at very early time points after Mtb infection (Fig. S12) but not later. Higher SIV RNA levels were observed in granulomas with viable Mtb compared with those without detectable Mtb and in the non-granulomatous lymph nodes (avoiding cellular distortion from granulomas) of relapsed animals compared to non-relapse animals (Fig. S8C). These data are consistent with our prior work showing discordance between systemic and localized immune responses (23) as well as the synergistic relationship between SIV and Mtb within tissues (17).

**TABLE 1:** Analytical summary of CD4 and CD8 T cell mycobacterial specific cytokine responses in granulomas from relapsed and non-relapsed animals^*a*^

	CD4	CD8
Frequency	NS	NS
IFN-γ	NS	NS
IL-2	NS	NS
TNF	NS	NS
IFN-α	NS	NS
IL-4	NS	NS
IL-10	NS	NS
IL-17	NS	NS
Gran B	NS	NS

^*a*^ NS, no significant difference between groups. See Fig. S9 for details.

**TABLE 2:** Analytical summary of CD4 and CD8 T cell frequencies and mycobacterial specific cytokine responses in bronchoalveolar lavage (BAL) from relapsed and non-relapsed animals^*a*^

	CD4			CD8		
	Mtb infection	Drug treatment	SIV induced relapse	Mtb infection	Drug treatment	SIV induced relapse
Frequency	NS	NS	NS	NS	NS	NS
IFN-γ	NS	NS	NS	NS	NS	NS
IL-2	NS	NS	**↑R**	NS	NS	NS
TNF	NS	NS	NS	NS	NS	NS
IL-10	NS	NS	NS	NS	NS	NS
IL-17	NS	NS	NS	NS	NS	NS

^*a*^ NS, no significant difference between groups; **­↑R**, significantly higher in the relapse groups. See Fig. S10 and S11 for details.

**TABLE 3:** Analytical summary of CD4 and CD8 T cell frequencies and mycobacterial specific cytokine responses in peripheral blood from relapsed and non-relapsed animals^*a*^

	CD4			CD8		
	Mtb infection	Drug treatment	SIV induced relapse	Mtb infection	Drug treatment	SIV induced relapse
Frequency	NS	NS	NS	NS	NS	NS
IFN-γ	**↓R**	NS	NS	NS	**↓R**	NS
IL-2	NS	NS	NS	NS	NS	NS
TNF	NS	NS	NS	NS	NS	**↓R**
Th1 Poly	NS	NS	NS	NS	NS	NS
Th1 any	NS	NS	NS	NS	NS	**↓R**
IL-10	NS	NS	NS	NS	NS	NS
IL-17	NS	NS	**↓R**	NS	NS	NS
CD107A	NS	NS	NS	NS	NS	NS
Gran B	**↓R**	NS	NS	NS	NS	NS
Granulysin	**↓R**	NS	NS	NS	**↓R**	NS
Perforin	NS	**↓R**	NS	**↓R**	NS	NS

^*a*^ NS, no significant difference between groups; **↓R**, significantly lower in the relapse groups. See Fig. S13 and S14 for details.

## DISCUSSION

Using our macaque model, we developed a model of TB relapse and characterized the events during the treatment of active TB and SIV-induced relapse. Although relapse was defined as a newly developed lesion after drug treatment, it was validated by gross pathology and bacterial burden at necropsy. We found that PET CT characteristics before drug treatment (Fig. 4) did not correlate with relapse outcome, and changes in inflammation (FDG avidity) or size of individual lesions during treatment were not associated with the presence or absence of viable Mtb at necropsy nor with the likelihood of dissemination occurring from a specific lesion. Our findings are consistent with human TB treatment trials in which no PET CT characteristics before, during, or at the end of shortened treatment could definitively predict relapse, and variable responses in size and metabolic activity of different lesion types were observed in the same host (reviewed in reference 27). To our knowledge, this is the first study to track bacterial dissemination during SIV-induced TB relapse. Using barcoded Mtb, we were able to harvest scan-matched lesions; correlate the timing of appearance during infection, treatment, and post-SIV; and track dissemination. Only 42% of barcodes from “pre-SIV” lesions could be detected in relapse lesions, demonstrating that not all granulomas with viable Mtb are capable of dissemination, even during acute SIV infection. This is consistent with our findings that granulomas function independently from one another in the same host (23, 24). Barcodes found in the relapse lesions could be traced to both lung granulomas and thoracic lymph node sites observed prior to SIV infection. In one case, a barcode from a relapsed lesion could only be traced to a thoracic lymph node, underscoring the importance of lymph nodes as a reservoir for relapse. Finally, one animal without relapse still had a low burden of Mtb in a granuloma. Thus, it stands to reason that the primary goal of TB treatment should be rapid and complete sterilization from all anatomic reservoirs, although some low-level persistence of Mtb may not result in clinical relapse. This is consistent with human studies where sputum or airway Mtb RNA occurred in the context of "non-resolving" or "intensifying" PET CT lesions observed at the conclusion of standard TB treatment, without evidence of relapse noted at the 1-year follow-up period (28).

To date, there are no reliable biomarkers that accurately predict TB cure, making efforts to shorten TB treatment a significant challenge. For example, in a recent 4-month treatment trial, relapse rates were unacceptably high (~20%), forcing the study to be stopped prematurely (29). Biomarkers associated with TB cure, including systemic immune markers (e.g., CD27, plasma cytokines) and microbiologic markers (e.g., sputum conversion), have had limited success (reviewed in reference 5). PET CT studies vary in patient population, drug regimen, immune status (HIV-infected vs. HIV-naïve), interval assessments after treatment, and imaging metrics (27). Using PET CT to monitor the treatment of drug-susceptible TB patients, 14% of the patients had a resolved pattern (no metabolic activity), but the remaining patients had either persistent metabolic activity or a mixed pattern (increased metabolic activity or new lesions). Although no failures were observed in the resolved group, treatment failure was observed in 28% of those with a mixed pattern (10). The risk of treatment failure was higher among patients who had less than an 80% reduction in total glycolytic activity index (a measure of lung metabolic activity via FDG) from the time of diagnosis to month 6 of treatment (10). Other studies focused on patients with HIV-TB coinfection have shown that using the maximum metabolic activity of involved lymph nodes (both pulmonary and extrapulmonary) was a better predictor of microbiologic response to therapy (27). Overall, it seems that the absence of residual lung metabolic activity by PET at the end of treatment (independent of HIV status) appears to be associated with cure, but residual metabolic activity is common and without predictive value for relapse (reviewed in reference 27).

There are several limitations to this study. The traditional patient treatment for active TB includes RIF, INH, pyrazinamide, and ethambutol for 2 months followed by 4 months of INH and RIF. We purposely chose a short course of RIF and INH to increase the probability of relapse as proof of principle, given the limited number of animals in this study. It is possible that differences in drug composition and duration could lead to alterations in immune responses or bacterial persistence in our model that may not be entirely translatable to humans. Since we had not performed relapse studies previously in this model, we were unable to perform a robust power analysis to determine an adequate sample size. Given the human relapse data, it is probable that large numbers of animals would be needed, which is neither practically nor ethically feasible. It is worth mentioning that limited statistical power increases the chance of type II error and that these findings should be validated in larger studies. It is possible that more subtle differences in the PET CT parameters or immunologic parameters would be identified as biomarkers for treatment failure in future studies. Nonetheless, the short-course treatment was effective in some animals, providing the opportunity to compare relapse and non-relapse in this study. Such relapse scenarios could reflect interrupted drug treatment or shortened treatment courses (30) in the context of HIV infection. Importantly, this study does not replicate the more common scenario of HIV-TB coinfection in which Mtb burden is likely to be much higher prior to TB treatment and where immune-driven efforts to sterilize Mtb are hindered by HIV-driven immune suppression.

In summary, we have developed a model of TB relapse in which the source of bacterial dissemination can be tracked and PET CT characteristics can be validated by bacterial burden at necropsy. This model could be useful in assessing new short-course treatment regimens and for gaining insights into the events that occur during relapse.

## SUPPLEMENTAL MATERIAL

Supplemental material iai.00177-25-s0001.pdf Tables S1 to S6; Fig. S1 to S20.

ASM does not own the copyrights to Supplemental Material that may be linked to, or accessed through, an article. The authors have granted ASM a non-exclusive, world-wide license to publish the Supplemental Material files. Please contact the corresponding author directly for reuse.
