# Supplementary material for: Characterizing PET CT patterns and bacterial dissemination features of tuberculosis relapse in the macaque model
Source: Infect Immun. 2025 Jun 23;93(8):e00177-25. doi: 10.1128/iai.00177-25 (PMC12341373; doi:10.1128/iai.00177-25)
Supplement: Supplemental material — Tables S1 to S6; Fig. S1 to S20. [file iai.00177-25-s0001.pdf]

	Total Animals	Age at Mtb Infection (Years)		Gender (Male)	Time to Treatment (Weeks)
	n	*Mean*	*SD*	*Percent*	*Mean*
**No Relapse**	4	7.1	1.1	100.0%	16.9
**Relapse**	8	7.0	0.7	87.5%	16.7

Supplementary Table 1. Distribution of age, gender, and time to drug treatment among the relapse and non-relapse animals.

Antibody	Clone	Compartment	Company
CD3	SP34	PBMC, BAL, Tissue	Pharmingen, San Diego, CA
CD4	L200	PBMC, BAL, Tissue	BD Horizons, Franklin Lakes, NJ
CD8	SK1	PBMC, BAL, Tissue	BD Biosciences, Franklin Lakes, NJ
CD163	GHI/61	BAL	BD Biosciences, Franklin Lakes, NJ
CD206	19.2	BAL	BD Biosciences, Franklin Lakes, NJ
CD11c	ICRF44	BAL	BD Biosciences, Franklin Lakes, NJ
CD45Ra	H100	PBMC	BD Biosciences, Franklin Lakes, NJ
CD27	M-T271	PBMC	BD Biosciences, Franklin Lakes, NJ
Granulysin	eBioDH2	PBMC	eBiosciences, Waltham, MA
Granzyme B	GB11	PBMC, Tissue	BD Biosciences, Franklin Lakes, NJ
CD107a	eBioH4A3	PBMC	eBiosciences, Waltham, MA
Perforin	pf344	PBMC	Mabtech, Nacka Strand Sweden
IFN-γ	B27	PBMC, BAL, Tissue	BD Biosciences, Franklin Lakes, NJ
IL-2	MQ1-17H12	PBMC, BAL, Tissue	BD Biosciences, Franklin Lakes, NJ
IL-10	JEs3-9D7	PBMC, BAL, Tissue	eBiosciences, Waltham, MA
IL-17	eBio64CAP17	PBMC, BAL, Tissue	eBiosciences, Waltham, MA
TNF	Mab11	PBMC, BAL, Tissue	BD Biosciences, Franklin Lakes, NJ
IL-4	8D4-8	Tissue	eBiosciences, Waltham, MA
IFN-alpha	LT27:295	Tissue	Miltenyi Biotec, Bergisch Gladbach, North Rhine-Westphalia, Germany

Supplementary Table 2. Antibodies used for flow cytometry assays.

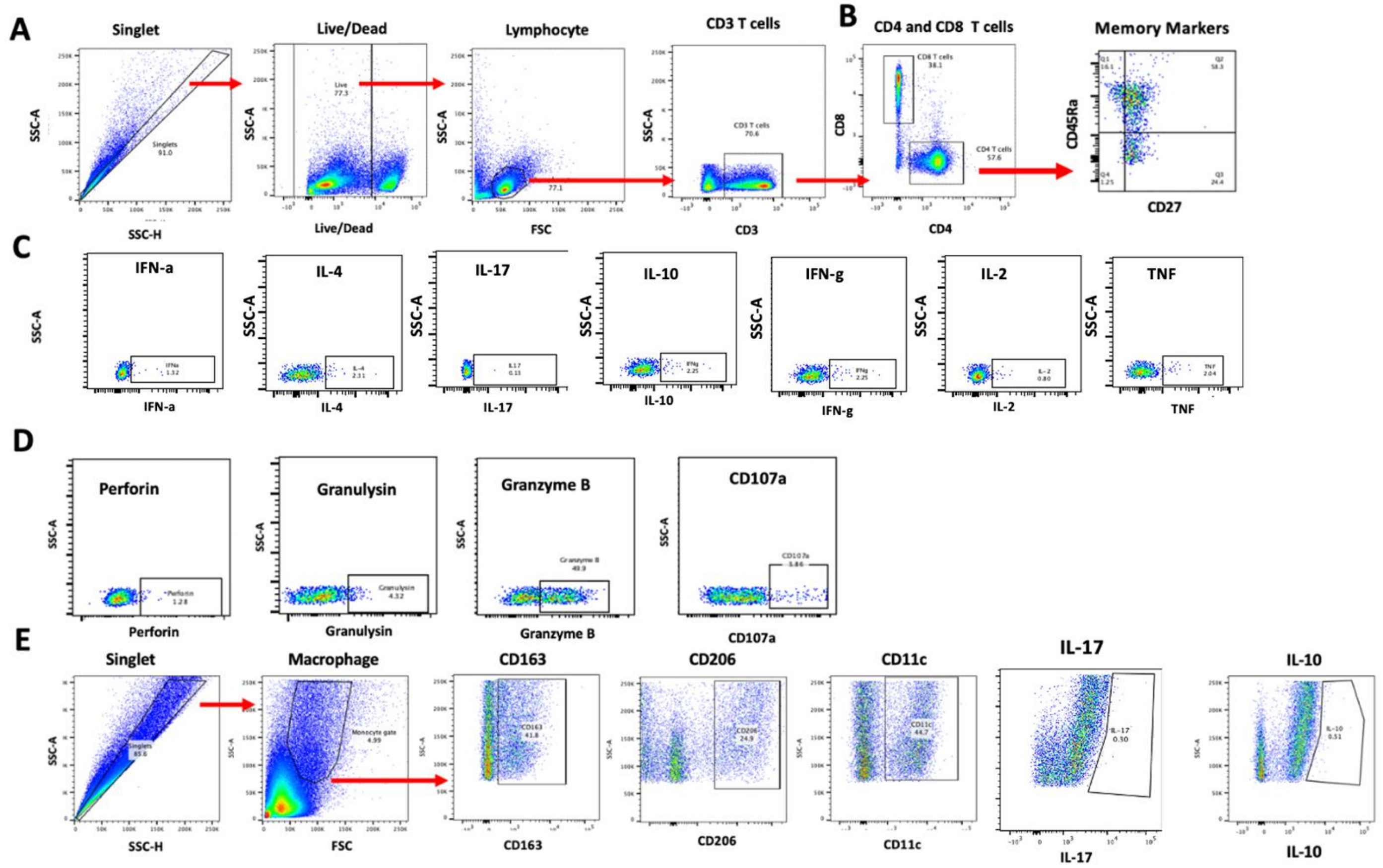

Supplementary Figure 1. **Example gating strategy for flow cytometry.** T cell gating strategy is the same for PBMC, BAL, and tissue samples. Macrophage gating is specific to BAL cells. A) PBMC stimulated with PDBU and Ionomycin. Singlets were positively selected. Live cells were selected out of the single cell gate. Lymphocytes were selected from the live cell gate. CD3 T cells were selected from lymphocytes and CD4 and CD8 T cells were selected from CD3 T cell gate. B) Memory Markers were selected from either CD4 or CD8 gate in PBMC. C) IFN-a, IL-4, IL-17, IL-10, IFN-g, IL-2, TNF were identified from the CD3 T cell gate. D) Perforin, Granulysin, Granzyme B, and CD107a were identified in CD3 T cells. E) Singlets were positively selected from BAL. Macrophage gate was selected from the singlet gate. CD163, CD206, CD11c, IL-17, and IL-10 from the monocyte gate.

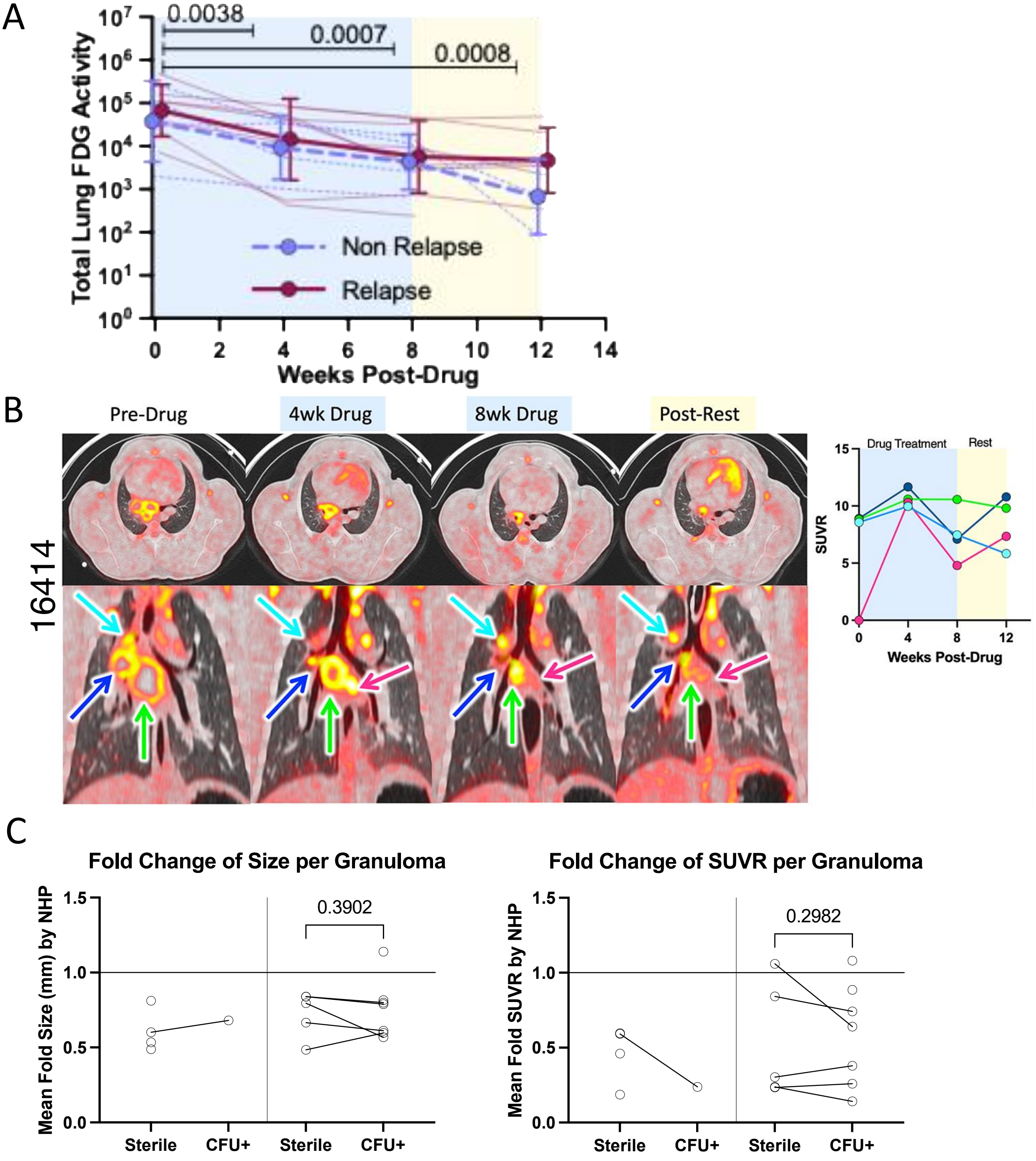

Supplementary Figure 2. Lung and thoracic lymph node inflammation during treatment. (A) Lung inflammation (FDG). A mixed-effects model showed that there was no difference between the two groups; however, FDG significantly decreased after drug treatment. Darker lines show mean and SD for each group; lighter lines represent trajectory of each animal. P values shown represent Dunnett's multiple comparison adjusted values comparing each time point to pre-drug treatment. **(B)** Independent and dynamic changes in metabolic activity among TB-associated thoracic lymph nodes (LN) before, during and after TB drug treatment. Axial (top row) and coronal images (lower row) of thoracic LN are shown. All lymph nodes increase in metabolic activity (standard uptake value ratio, SUVR) between 0 and 4 weeks-treatment, but diverge in pattern over time. Color-matched thoracic LN are shown in the coronal views (bottom row) and line plots (right) over time. **(C)** Change in granuloma size and avidity during drug treatment does not reflect bacterial burden. (Left) Average fold changes in size (mm) in sterile and CFU+ granulomas matched per animal. (Right) Average fold changes in avidity (SUVR) in sterile and CFU+ granulomas matched per animal. Each dot is the mean of all changes of granulomas per animal of either sterile (left) or CFU+ (right) granulomas. Lines connect averages per each individual animal. Only one animal in the non-relapse group had a granuloma that grew CFU. Two animals in the relapse group had no sterile granuloma that could be confidently analyzed on PET CT.

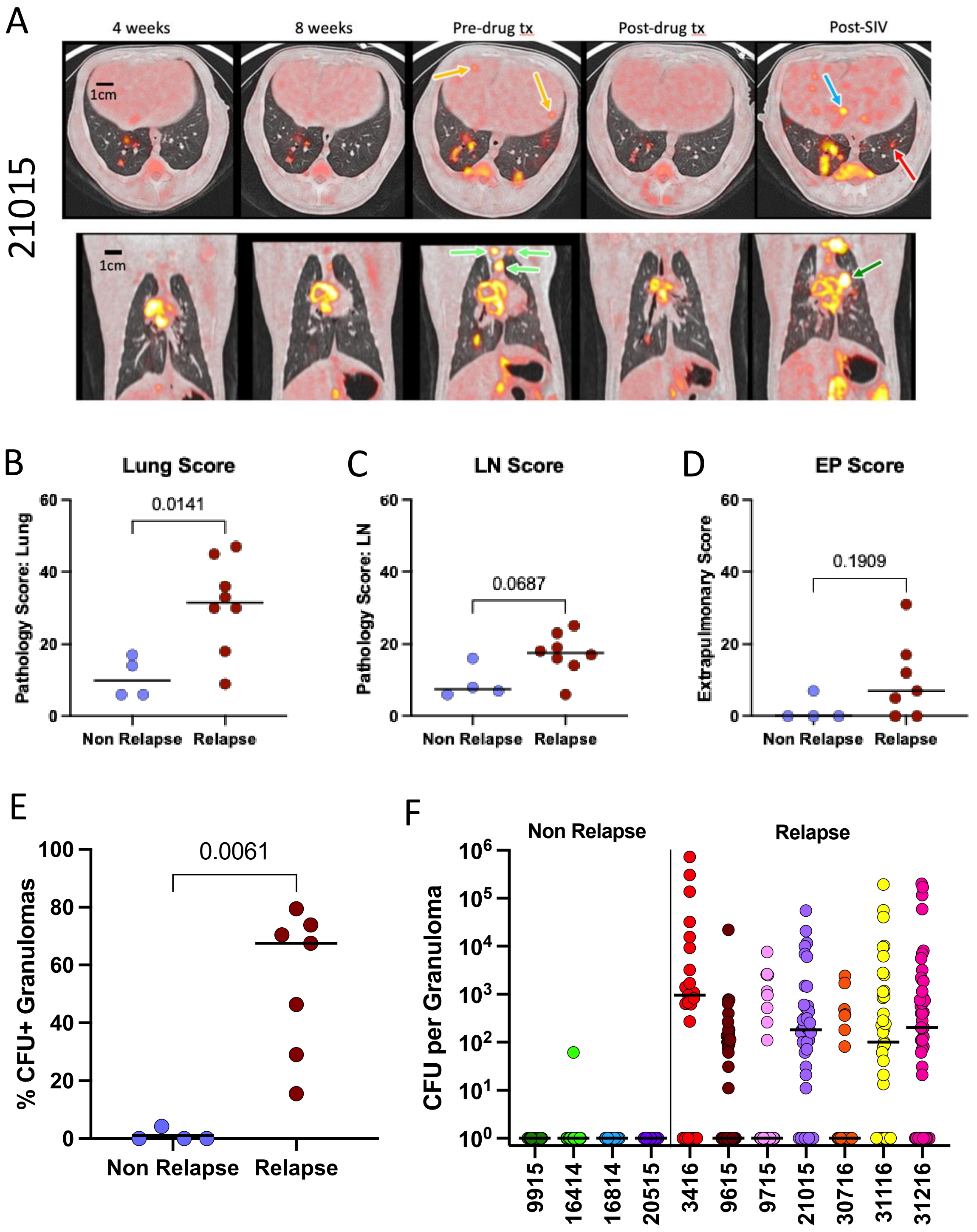

Supplementary Figure 3. PET CT evidence of SIV-induced relapse after TB drug treatment. (A) PET CT evidence of SIV-induced relapse after TB drug treatment. Top row: Synchronized axial images show liver granulomas before drug treatment (orange arrows) that resolve on subsequent scans, but new granulomas are seen post-SIV (blue arrow). While granulomas and clusters can be seen on all scans prior to SIV, there is a new granuloma (red arrow) in the left lower lobe. Bottom row: Coronal images show thoracic lymph node involvement in the upper mediastinum before drug treatment (light green arrows) and new post-SIV (dark green arrow). TB involvement of post-SIV lymph node was confirmed at necropsy. (B) PET CT defined relapse is associated with higher lung pathology and a trend to higher thoracic lymph node (LN) pathology (C). (D) Extrapulmonary score (derived from gross pathology and Mtb growth) was similar between relapse and non-relapse animals. (E) Relapse animals had a greater proportion of lung granulomas with Mtb growth. (F) Relapse animals have greater bacterial burden per lung granuloma (measured as colony forming units, CFU) compared to non-relapse animals. P-values shown reflect Mann-Whitney test. Each dot represents an animal, and lines represent medians.

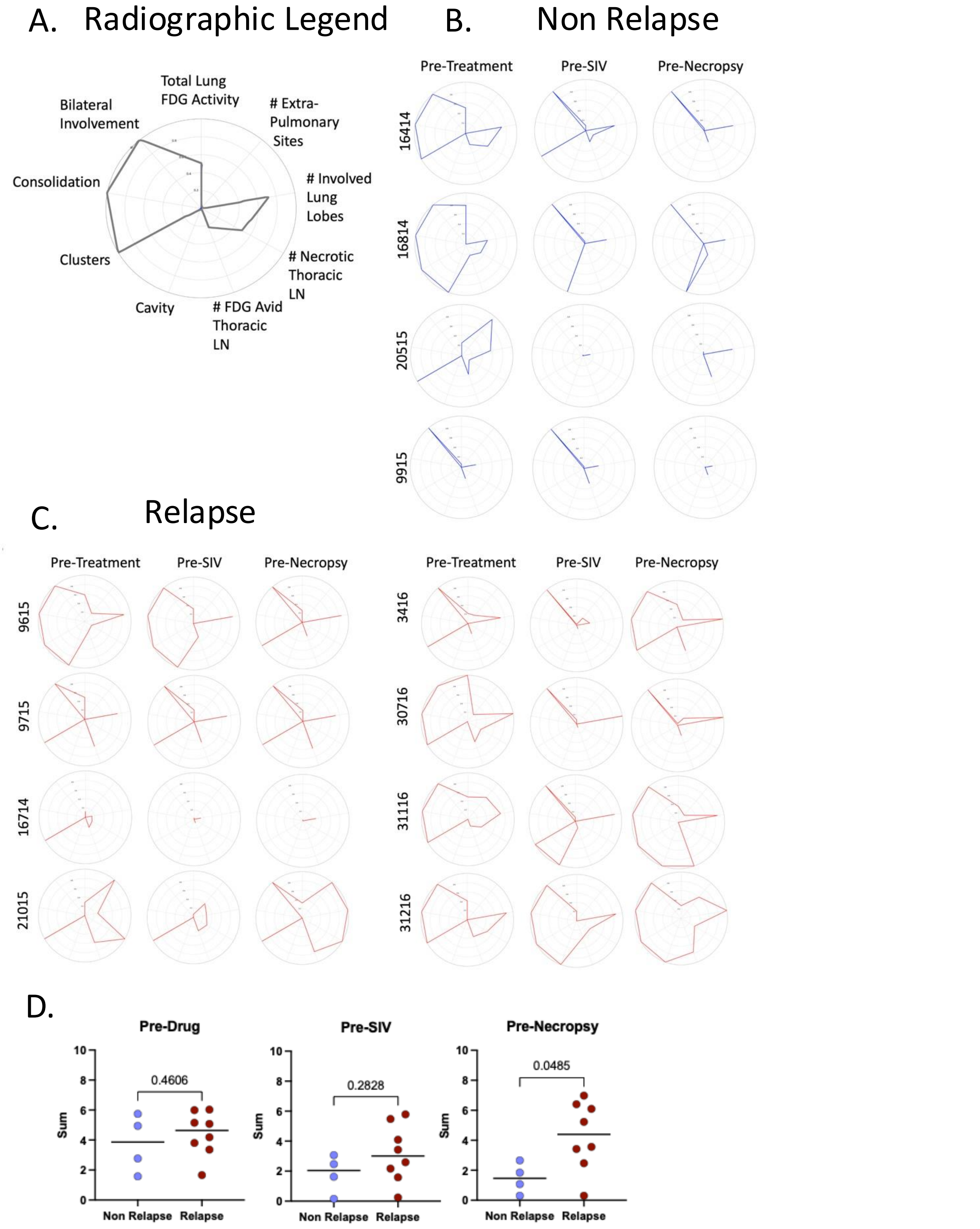

Supplementary Figure 4. Combined features of TB disease shown as spider plots. (A) Radiographic features of TB disease in composite with each radial coordinate measuring the severity of involvement. Composite of PET CT identified features of tuberculosis prior to treatment, after TB drug treatment but before SIV infection, before necropsy among animals that (B) did not relapse (B) and those that did relapse (C). FDG: F^{18} Fluoro-DeoxyGlucose, EP: Extra-Pulmonary, LN: Lymph Nodes. D) The sum of the radiographic feature scores were compared between relapse and non-relapse animals at serial time points Each dot represents an animal, lines represent medians. Mann-Whitney p-values shown.

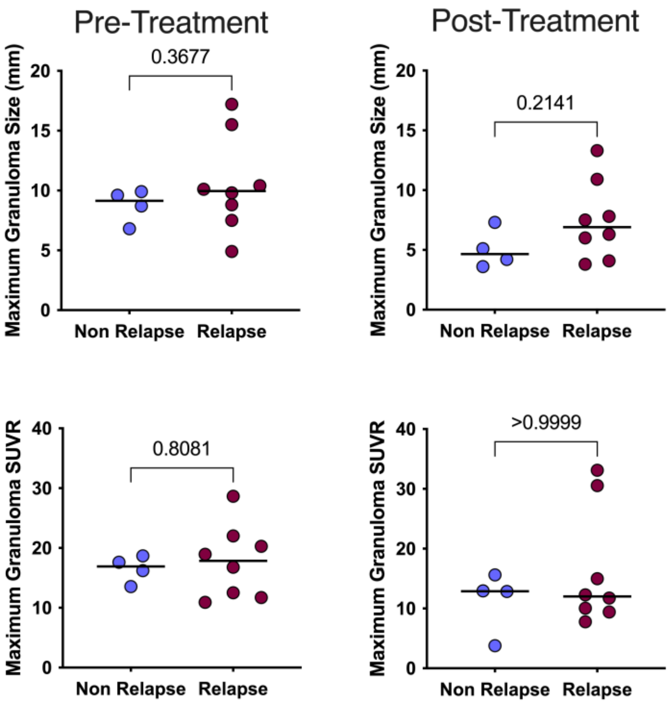

Supplementary Figure 5. PET CT features before and during drug treatment among relapsed (N=8) and non-relapsed (n=4) animals. Maximum size and metabolic activity (measured standard uptake value ratio, SUVR) for each animal before and after drug treatment. Each dot represents the maximum value for each animal, lines represent medians. P-values reflect Mann-Whitney analysis.

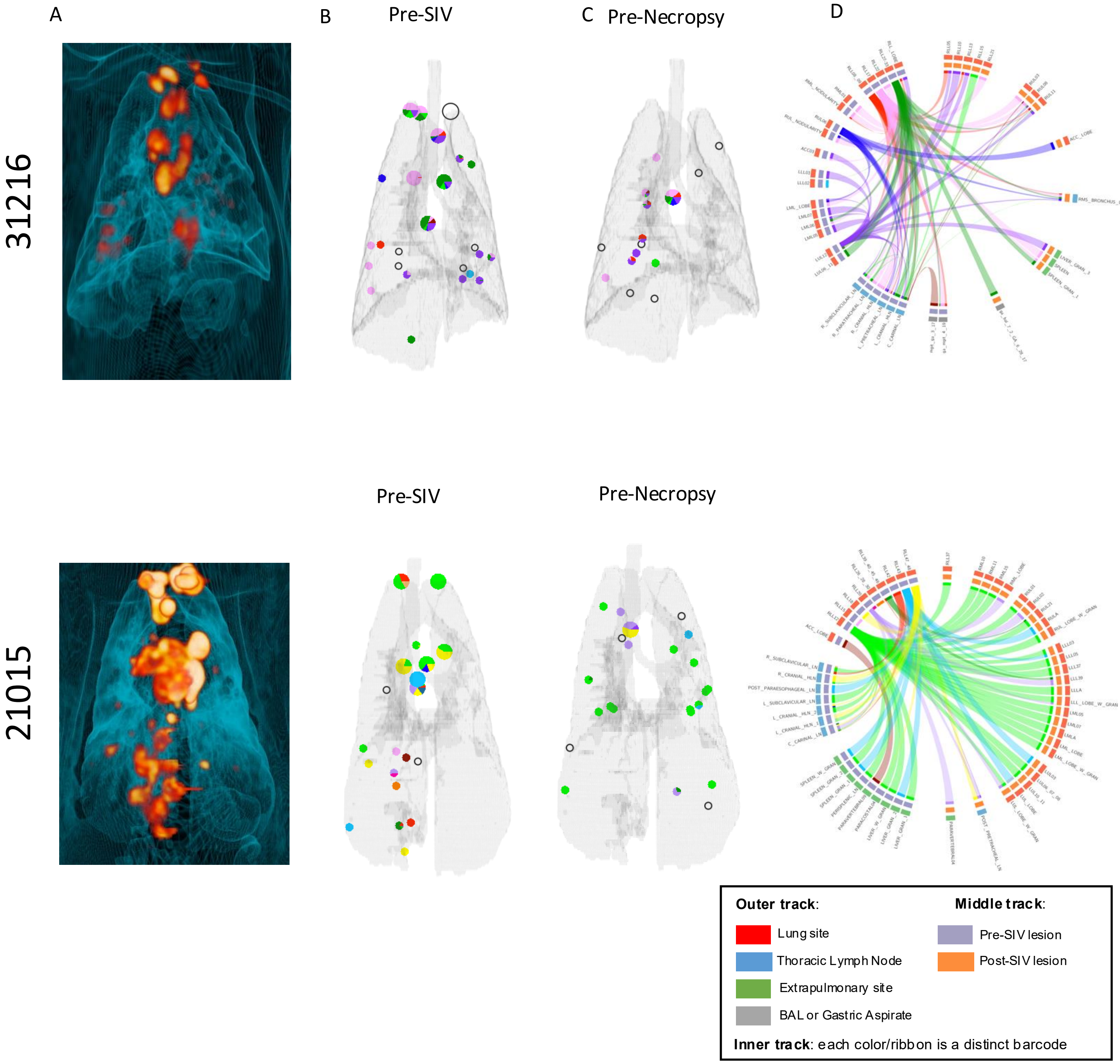

Supplementary Figure 6. Barcode distribution of disease for animals 31216 (top row) and 21015 (bottom panel). A) Three-dimensional volume rendering of lung disease pre-necropsy scan. (B) Anatomic distribution of Mtb barcodes from pre-existing granulomas and thoracic lymph nodes seen on scan before SIV-infection (B) and new granulomas and lymph nodes observed after SIV infection (pre-necropsy) (C). Open circles represent sterile tissue (B and C). Smaller circles represent granulomas or clusters and larger circles represent thoracic lymph nodes. (D) Circos plots representing barcoded Mtb detected in tissues (inner track), with ribbons representing tissues containing the same barcodes. Middle track represents timing of sample seen on scans (pre- or post- SIV infection) and outer track represents the anatomical compartment associated with each tissue. Distinct barcodes are represented by a different color, matched across B, C, and D.

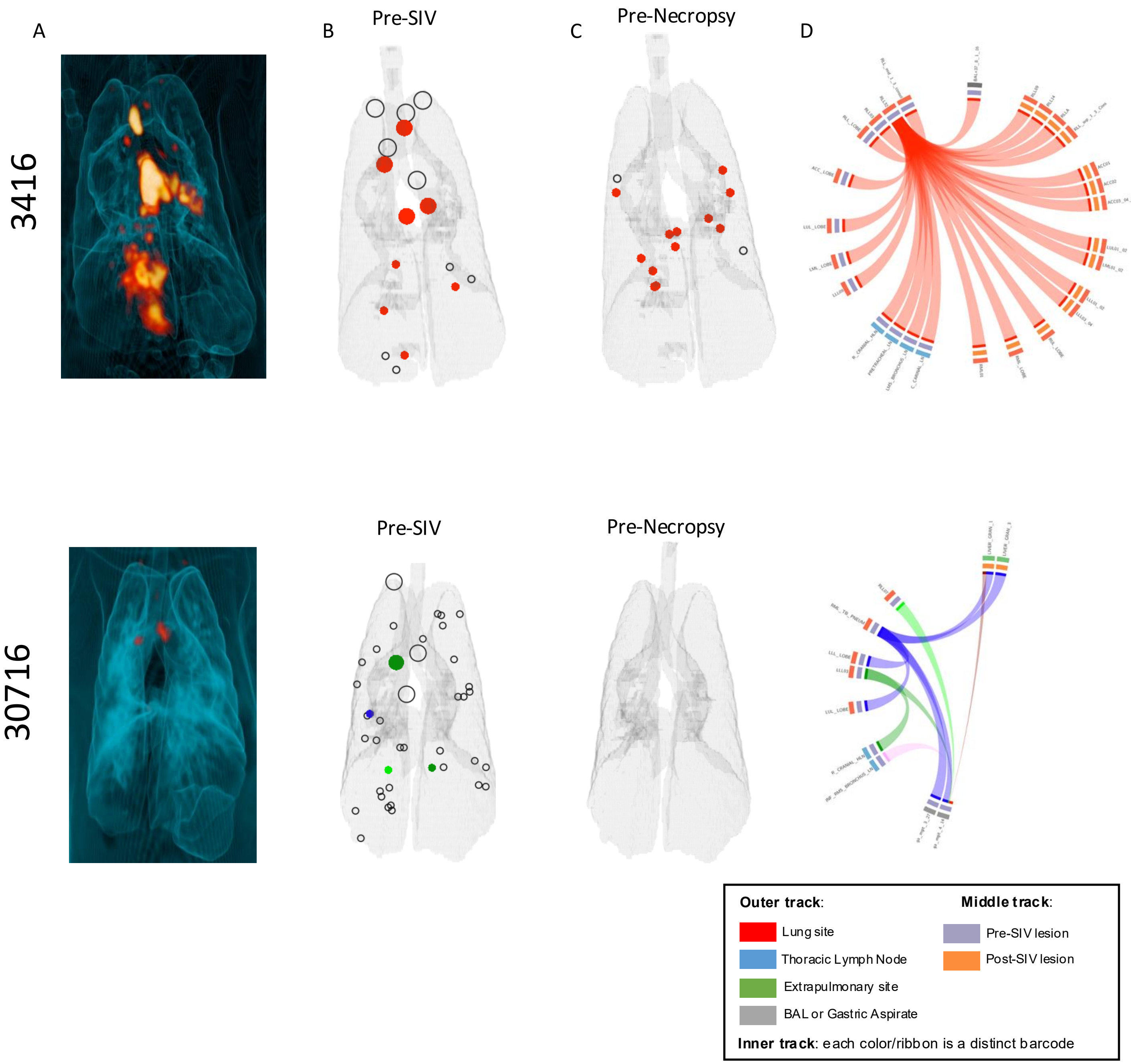

Supplementary Figure 7. Barcode distribution of disease for animals 3416 (top row) and 30716 (bottom panel). A) Three-dimensional volume rendering of lung and lymph node disease on pre-necropsy scan. (B) Anatomic distribution of barcodes found in granulomas and thoracic lymph nodes seen on scan before SIV-infection. (C) Distribution of new granulomas and lymph nodes seen on scan after SIV-infection (pre-necropsy). Open circles represent sterile tissue (B and C). Smaller circles represent granulomas or clusters and larger circles represent thoracic lymph nodes. (D) Circos plots representing barcoded Mtb detected in tissues (inner track), with ribbons representing tissues containing the same barcodes. Middle track represents timing of sample seen on scans (pre- or post- SIV infection) and outer track represents the anatomical compartment associated with each tissue. Distinct barcodes are represented by a different color, matched across B, C, and D.

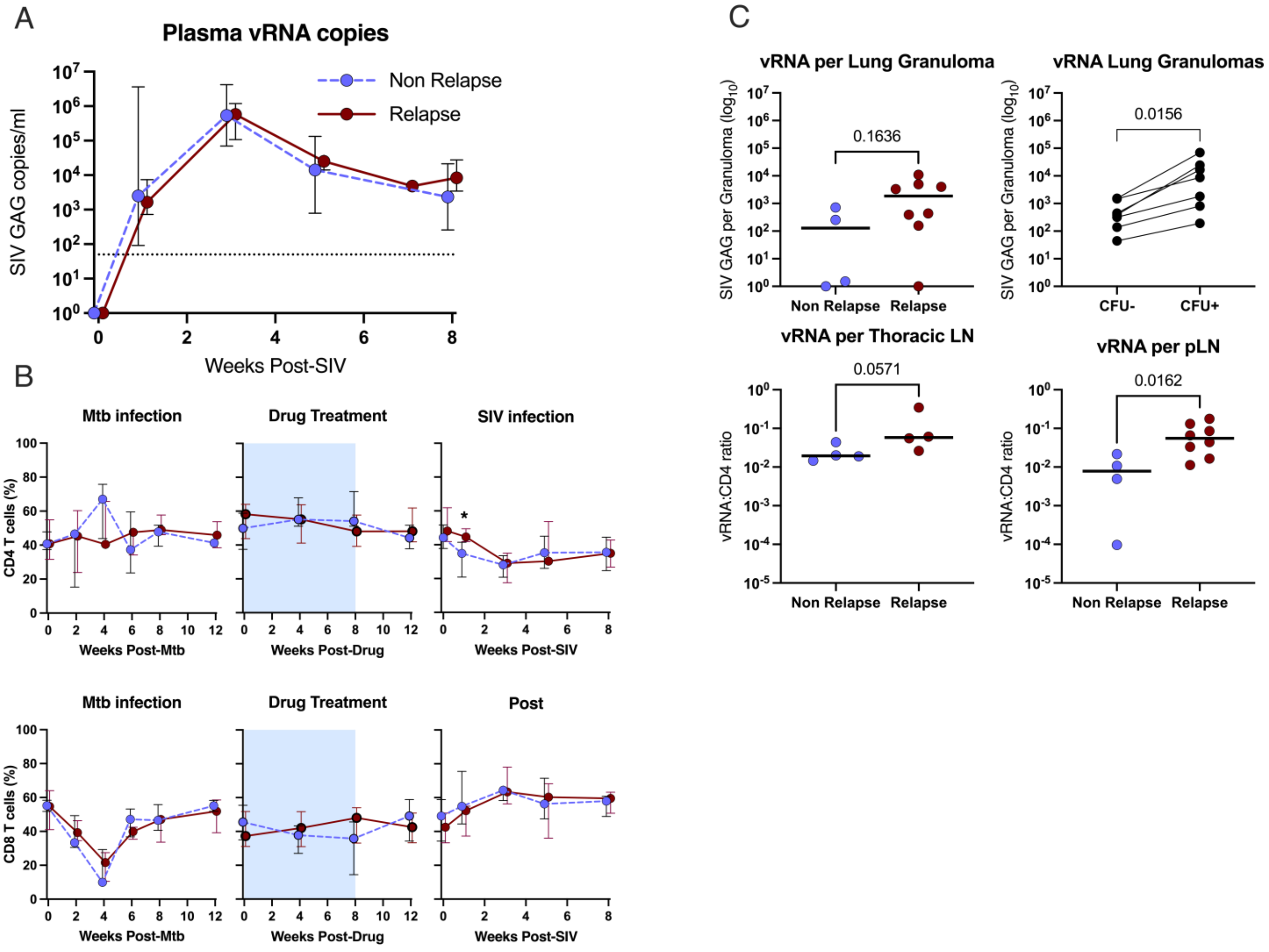

Supplementary Figure 8. Peripheral plasma RNA levels and CD4 T cells frequencies do not correlate with relapse. (A) SIV plasma RNA copies/ml are shown over time among relapse and non-relapse animals during SIV infection. (B) Frequency of peripheral blood CD4 and CD8 T cells within the CD3 gate during Mtb infection (left column), drug treatment (blue background) with drug free period (middle columns), and during SIV infection (right column). Medians with IQR are shown. Mann-Whitney tests were run at each time point with no correction for multiple comparisons. $p < 0.05$: *. (C) The median SIV RNA levels in granulomas was similar between relapse and non-relapse animals. Greater median SIV RNA levels were observed from granulomas with viable Mtb (CFU+) compared to those without viable Mtb (CFU-). Greater SIV RNA:CD4 ratios are observed in relapse animals in both thoracic and peripheral lymph nodes (pLN). Top row: Each dot represents the median SIV/CD4 RNA ratio per animal. Bottom row: Each dot represents an individual lymph node. For unpaired group comparisons, p-value determined by Mann-Whitney. For paired data, p-value is determined by Wilcoxon matched-pairs signed rank test. A and B) 8 animals in relapse group, 4 animals in non-relapse group (not all animals represented at each time point).

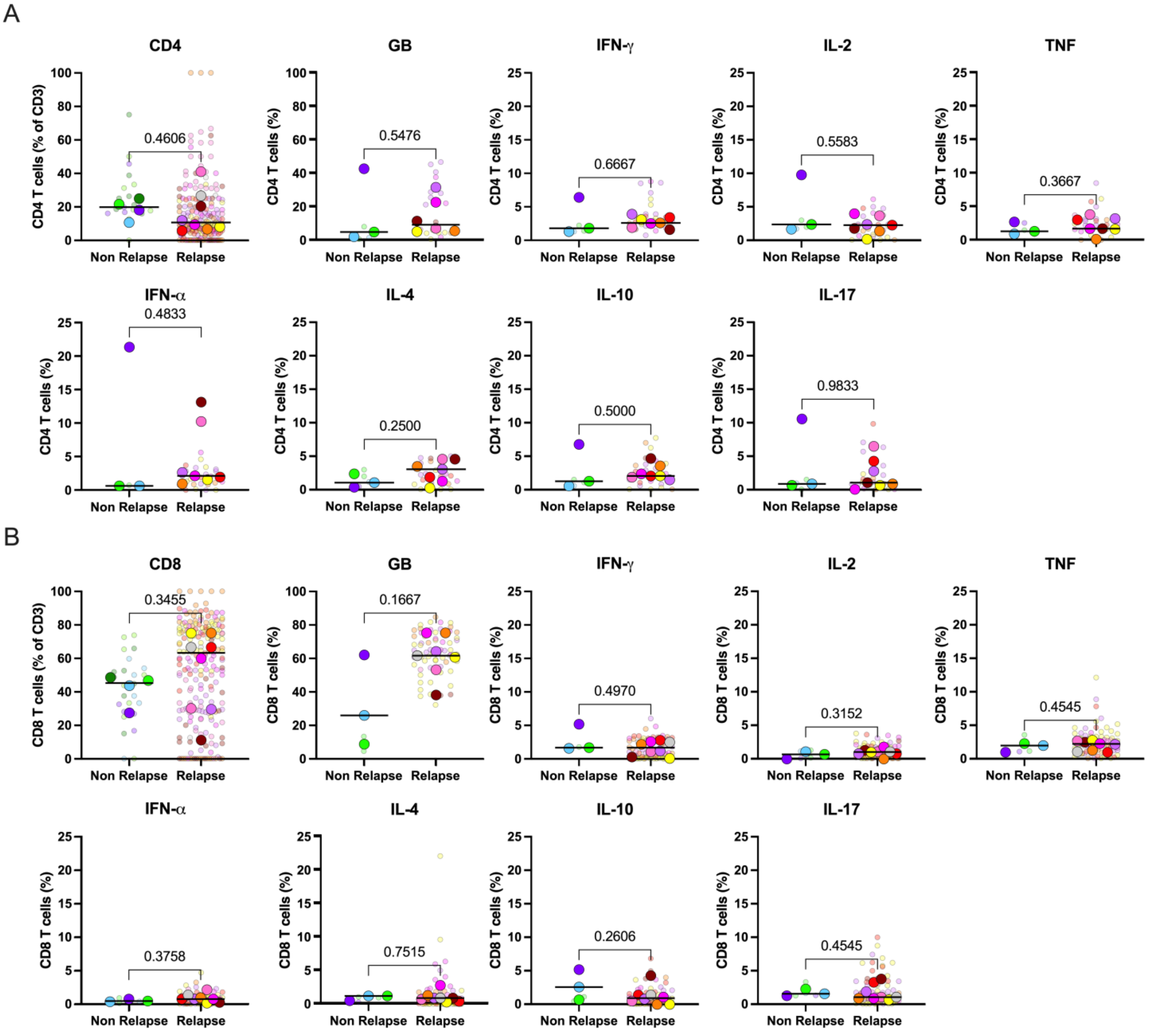

Supplemental Figure 9. **Mycobacterial specific CD4 (A) and CD8 (B) T cell responses from granulomas are similar between relapse (n=3-4) and non-relapse animals (n=6-8).** Small, transparent circles represent individual granulomas; large circles reflect the median functional response from all granulomas analyzed per animal. Circles are colored by animal, and lines represent medians. P-values reflect Mann-Whitney analysis. (GB=granzyme B)

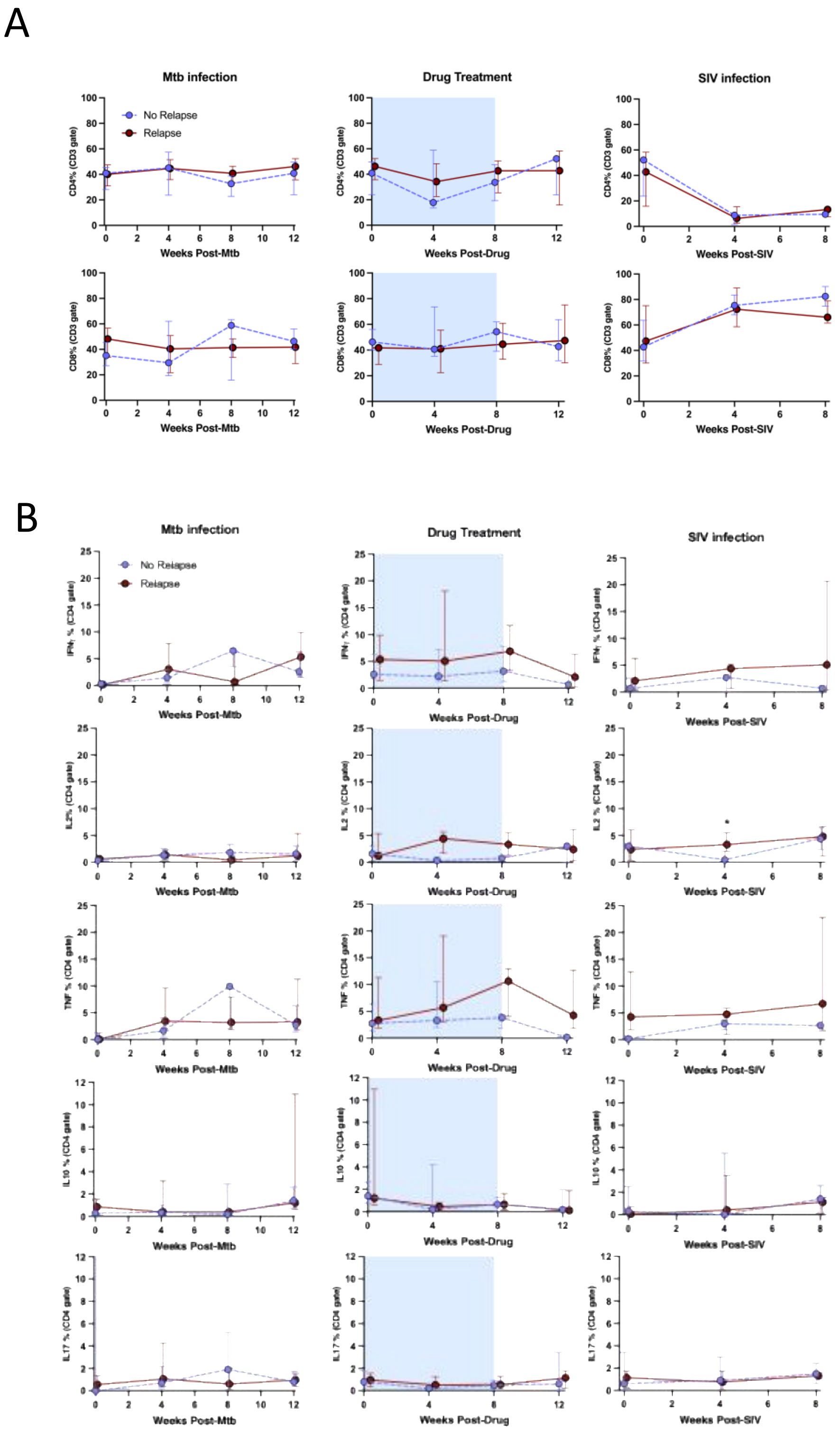

Supplementary Figure 10. (A) Serial frequencies of CD4 and CD8 T cells in the airways. Serial CD4 (top) T cell frequencies and serial CD8 (bottom) T cell frequencies in the airway are shown during Mtb infection, drug treatment, and SIV infection among relapse (n=4) and non-relapse animals (n=8). (B) Serial frequencies of Th1 (IFN-γ, IL-2, TNF), IL-10-, and IL-17-producing CD4 T cells in the airways during Mtb infection, drug treatment, and SIV infection among relapse (n=4) and non-relapse animals (n=8). Blue shaded area shows weeks of drug treatment, medians shown with IQR. Mann-Whitney tests were run at each time point with no correction for multiple comparisons. $0.05 < p < 0.10$: #, $p < 0.05$: *.

A

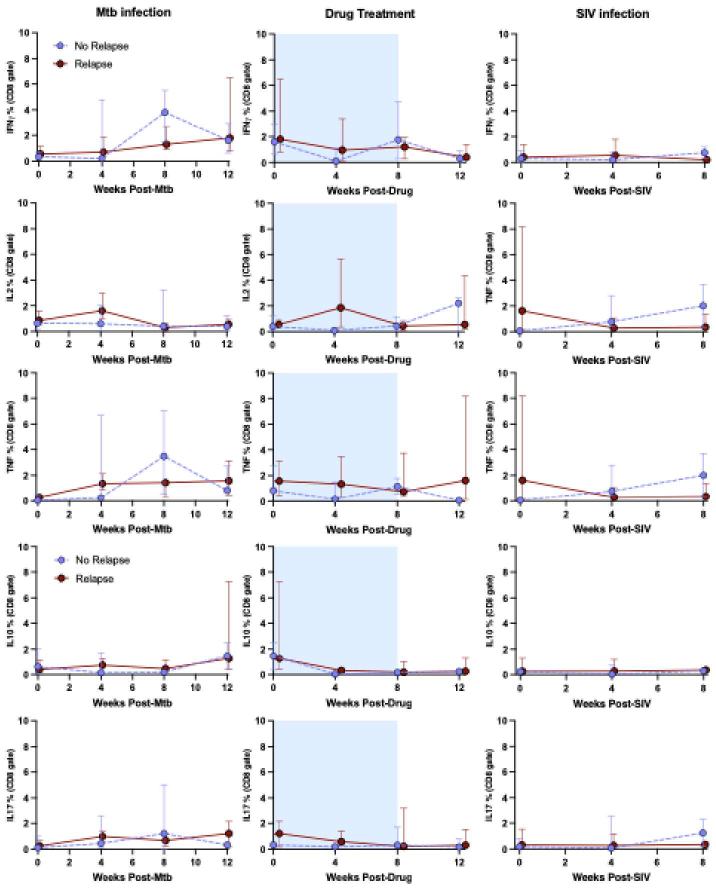

B

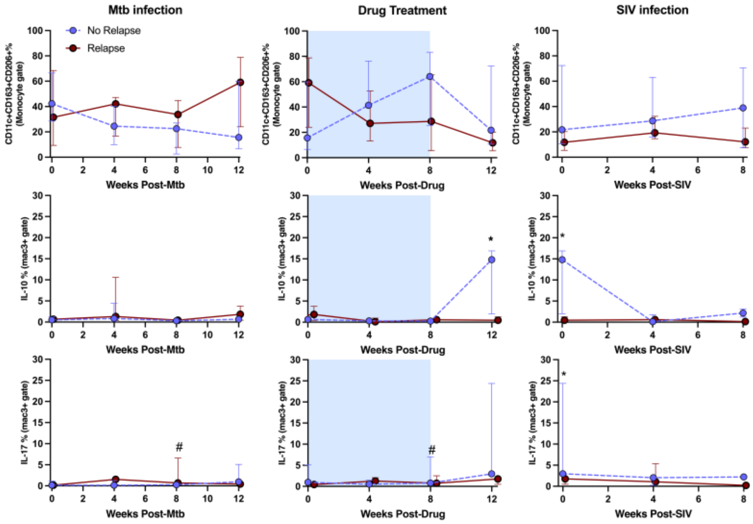

Supplementary Figure 11. (A) Serial frequencies of Th1 (IFN-γ, IL-2, TNF), IL10-, and IL17-producing CD8 T cells in the airways during Mtb infection, drug treatment, and SIV infection among relapse (n=4) and non-relapse animals (n=8). (B) Alveolar macrophage response in the airways over time. Top row: serial frequencies of alveolar macrophages (defined as CD11c+CD163+CD206+) in the airway. Middle row and bottom rows: Frequency of IL-10 and IL-17 producing alveolar macrophages over time. Blue shaded area shows weeks of drug treatment, medians shown with IQR Mann-Whitney tests were run at each time point with no correction for multiple comparisons. $0.05 < p < 0.10$: #, $p < 0.05$: *.

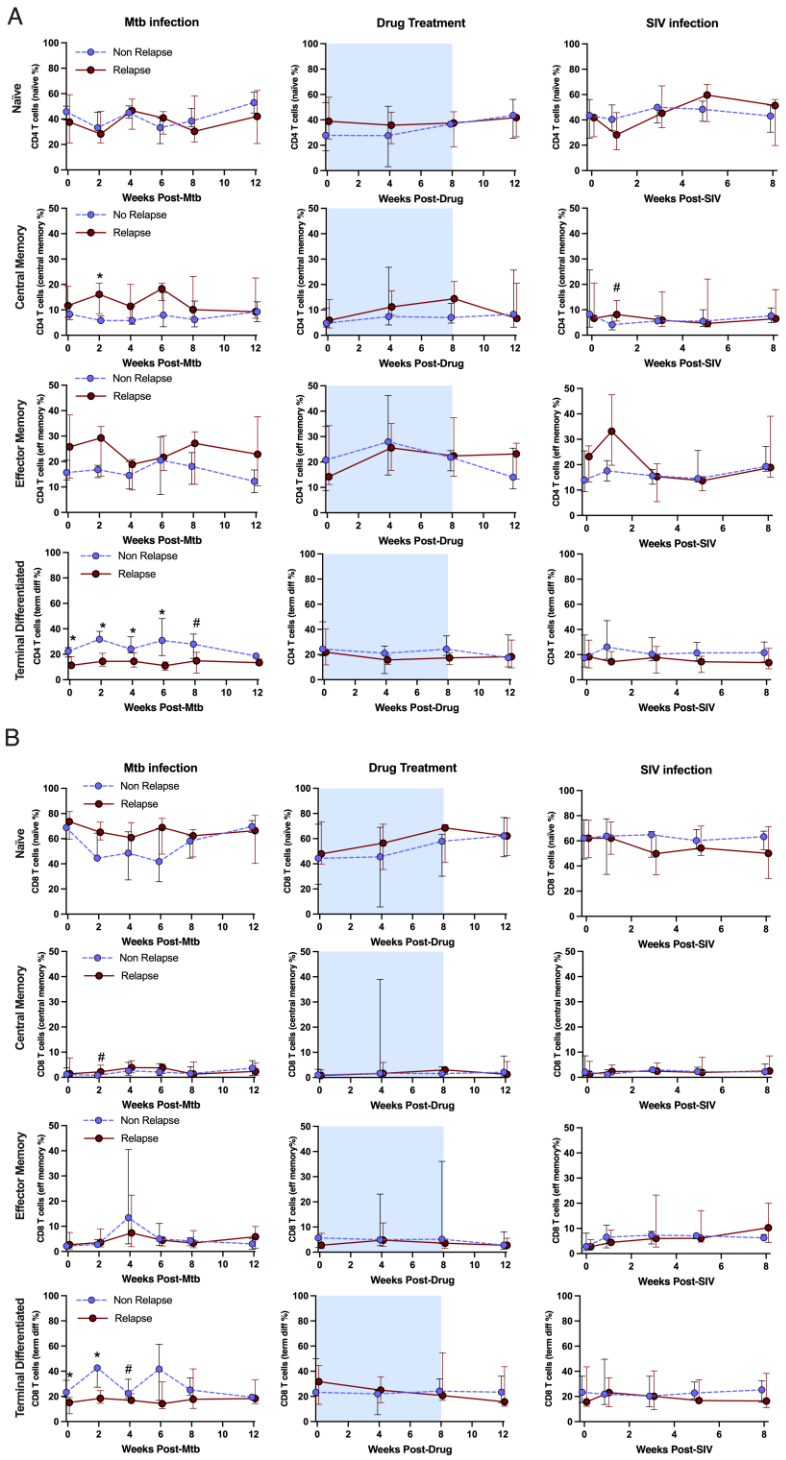

Supplementary Figure 12. (A) Distribution of CD4 T cells in peripheral blood mononuclear cell by memory type during Mtb infection, drug treatment with drug free period, and SIV infection among relapse (n=8, n=7 for drug treatment graphs) and non-relapse (n=4) animals. Naïve (CD27+CD45Ra+), Central memory (CD27+CD45Ra-), Effector memory (CD27-CD45Ra-), Terminal differentiated (CD27-CD45Ra+). A lower frequency of terminal differentiated CD4 T cells appears during the first 6 weeks of Mtb infection among animals that would later develop relapse compared to those without relapse. (B) Distribution of CD8 T cells in peripheral blood mononuclear cell by memory type during Mtb infection, drug treatment with drug free period, and SIV infection among relapse (n=8) and non-relapse (n=4) animals. A lower frequency of terminal differentiated CD8 T cells appears during the first 2 weeks of Mtb infection among animals that would later develop relapse compared to those without relapse. Blue background in middle panels indicates drug treatment. Each dot reflects the median frequency and IQR shown. Mann-Whitney test used to compare groups at each time point. Unadjusted p-values: $0.05 < p < 0.10$: #, $p < 0.05$: *.

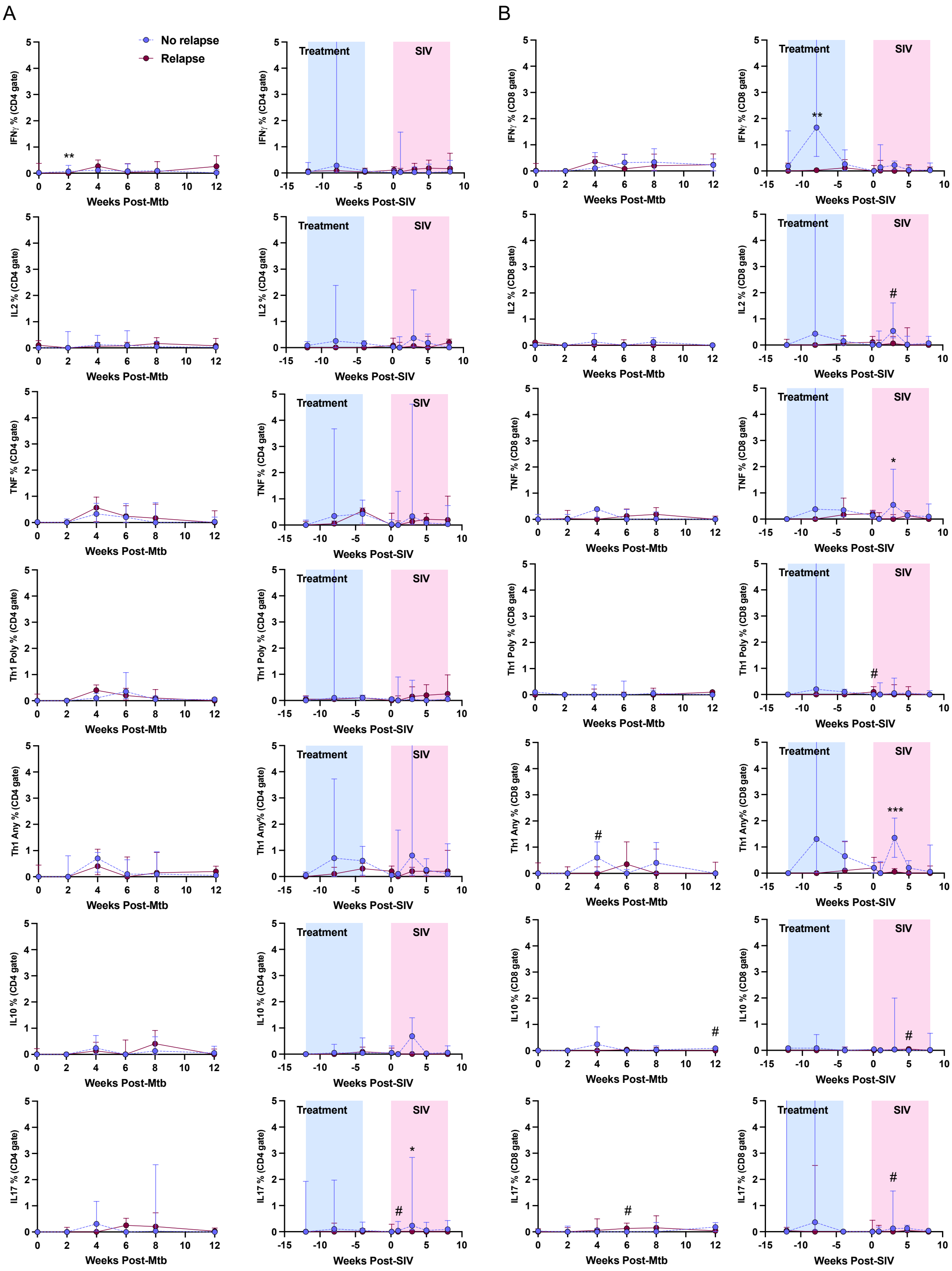

Supplementary Figure 13. Serial frequencies of Mtb-specific, Th1 (IFN-γ, IL-2, TNF), IL-10-, and IL-17-producing CD4 (A) and CD8 (B) T cells in the blood during Mtb infection, drug treatment, and SIV infection among non-relapse (n=4) and relapse (n=8) animals. Th1 Poly represents CD4 or CD8 T cells that produce two or more Th1 cytokines. Th1 Any represents CD4 or CD8 T cells that make at least one Th1 cytokine. Blue shaded area shows weeks of drug treatment, pink shaded area shows SIV-infection, medians shown with IQR. Mann-Whitney tests were run at each time point with no correction for multiple comparisons. $0.05 < p < 0.10$: #, $p < 0.05$: *.

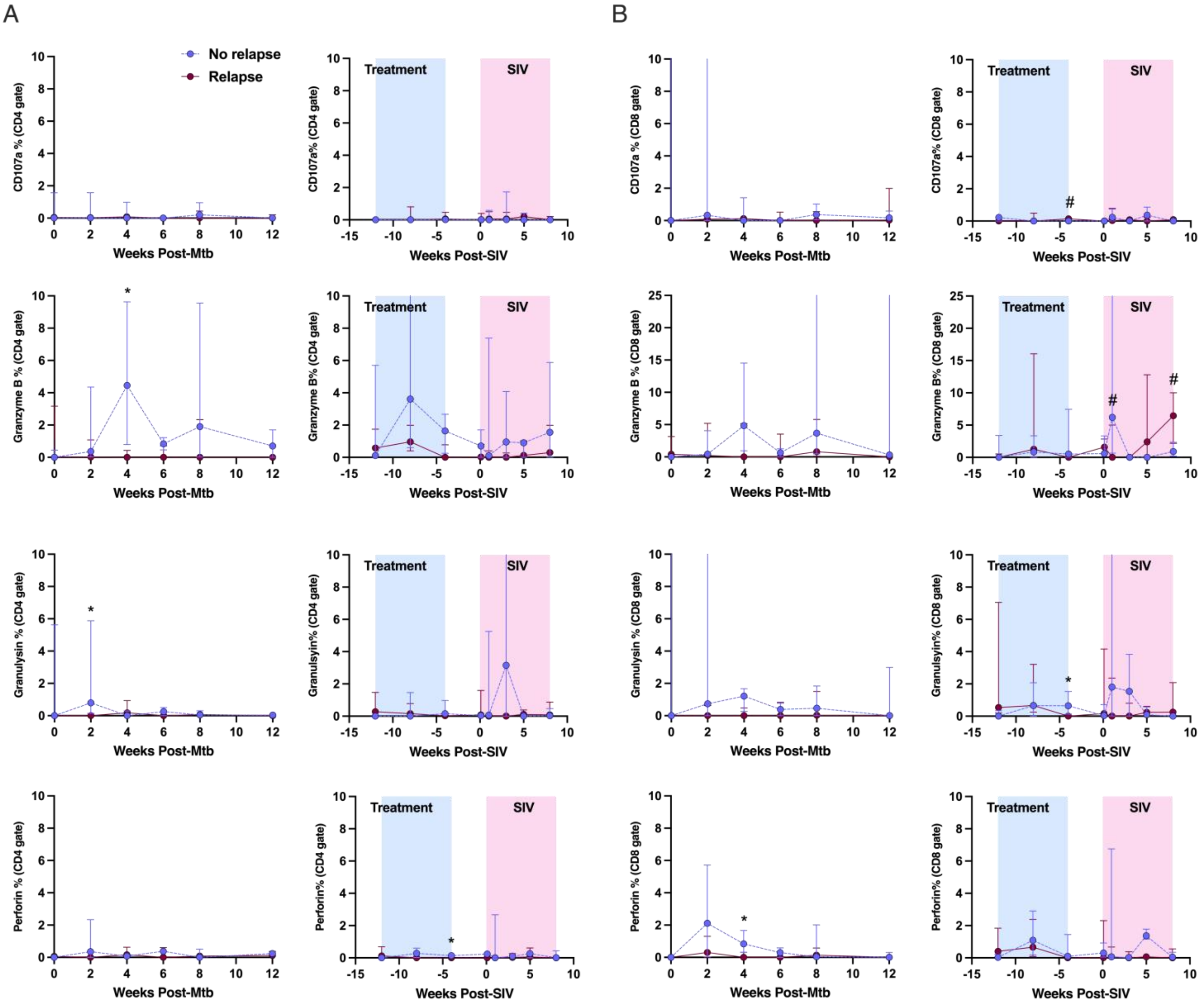

Supplementary Figure 14. Serial frequencies of Mtb-specific CD4 (A) and CD8 (B) T cells with cytolytic characteristics in the blood during Mtb infection, drug treatment, and SIV infection among non-relapse (n=4) and relapse (n=8) animals. Blue shaded area shows weeks of drug treatment, pink shaded area shows SIV-infection, medians shown with IQR. Mann-Whitney tests were run at each time point with no correction for multiple comparisons. $0.05 < p < 0.10$: #, $p < 0.05$: *.

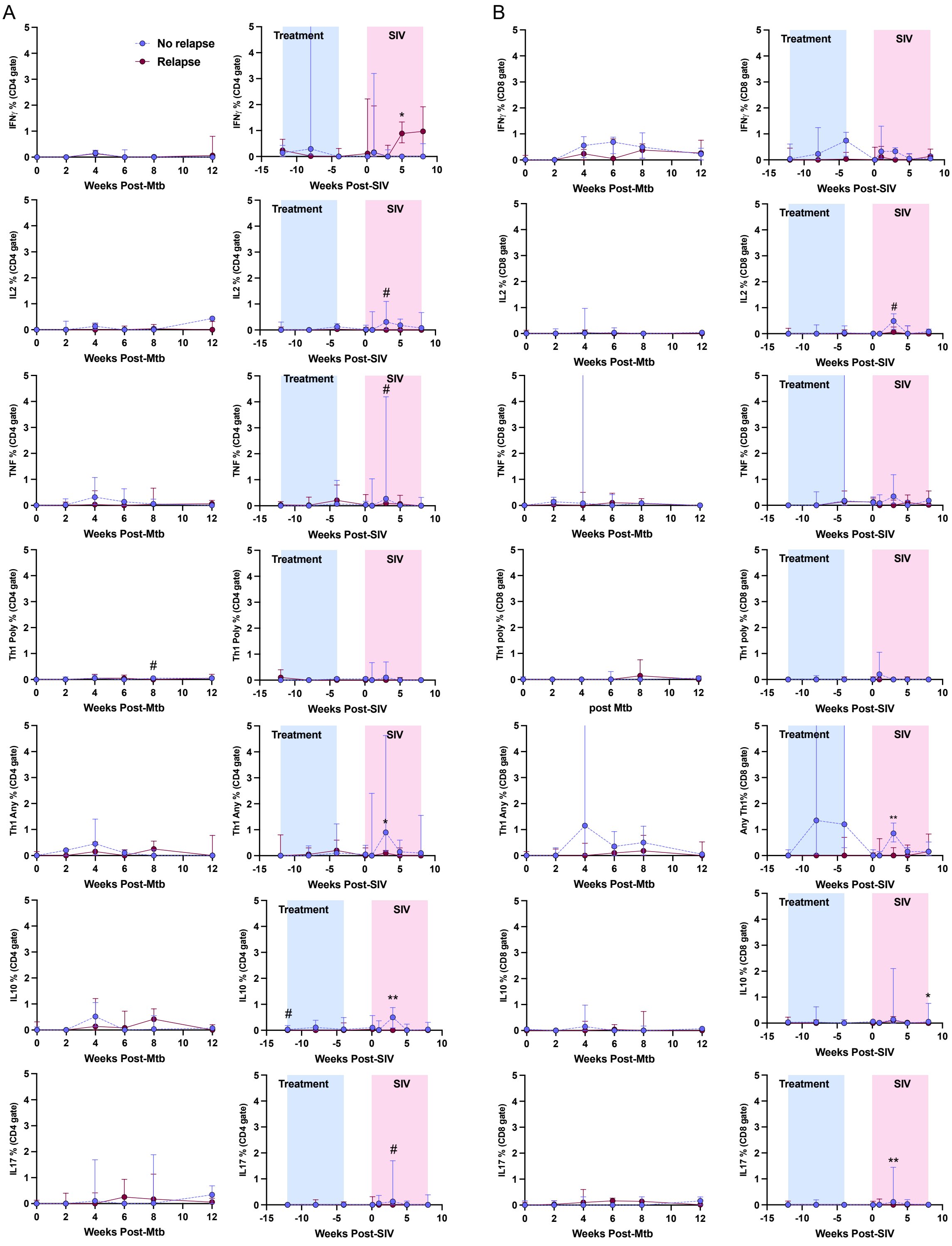

Supplementary Figure 15. Serial frequencies of Mtb-specific, naïve (CD45Ra+CD27+), Th1-, IL10-, and IL17-producing CD4 (A) and CD8 (B) T cells in the blood during Mtb infection, drug treatment, and SIV infection among non-relapse (n=4) and relapse (n=8) animals. Th1 Poly represents CD4 or CD8 T cells that produce two or more Th1 cytokines. Th1 Any represents CD4 or CD8 T cells that make at least one Th1 cytokine. Blue shaded area shows weeks of drug treatment, pink shaded area shows SIV-infection. Mann-Whitney tests were run at each time point with no correction for multiple comparisons. 0.05 < p < 0.10: #, p < 0.05: *.

A. Naïve Cytolytic

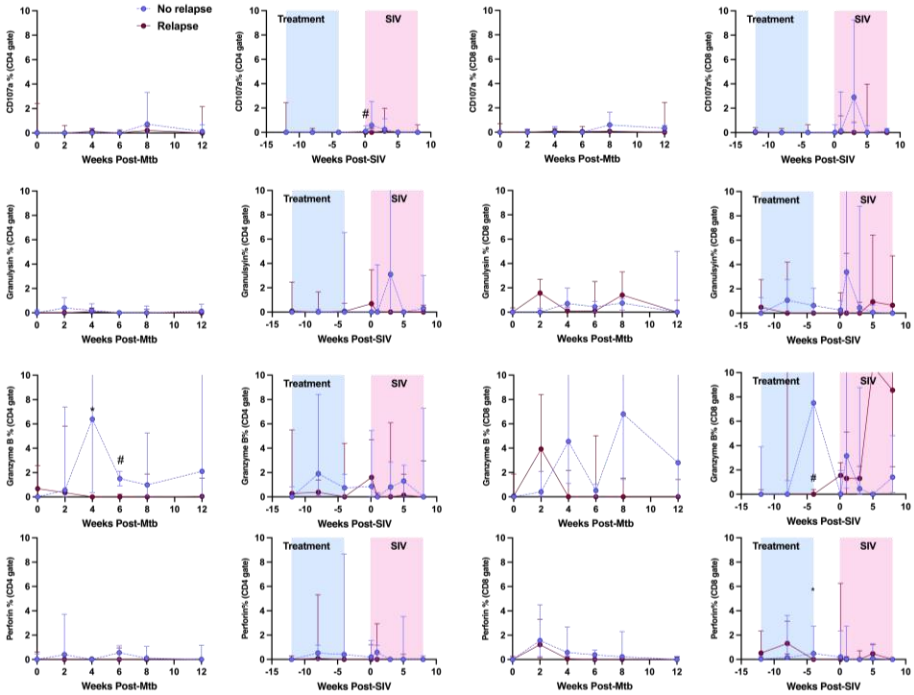

B. Central Memory Cytolytic

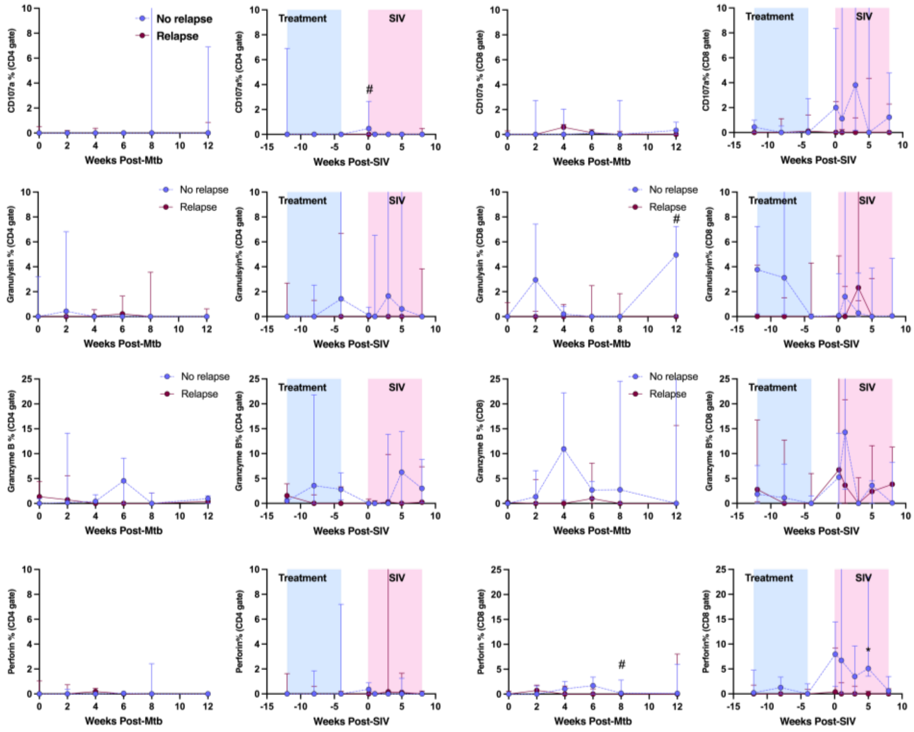

Supplementary Figure 16. (A) Serial frequencies of Mtb-specific, naïve (CD45Ra+CD27) CD4 and CD8 T cells with cytolytic characteristics in the blood during Mtb infection, drug treatment, and SIV infection among non-relapse (n=4) and relapse (n=8) animals. (B) Serial frequencies of Mtb-specific, central memory (CD45Ra-CD27+) CD4 and CD8 T cells with cytolytic characteristics in the blood during Mtb infection, drug treatment, and SIV infection among non-relapse (n=4) and relapse (n=8) animals. Blue shaded area shows weeks of drug treatment, pink shaded area shows SIV-infection, medians shown with IQR. Mann-Whitney tests were run at each time point with no correction for multiple comparisons. $0.05 < p < 0.10$: #, $p < 0.05$: *.

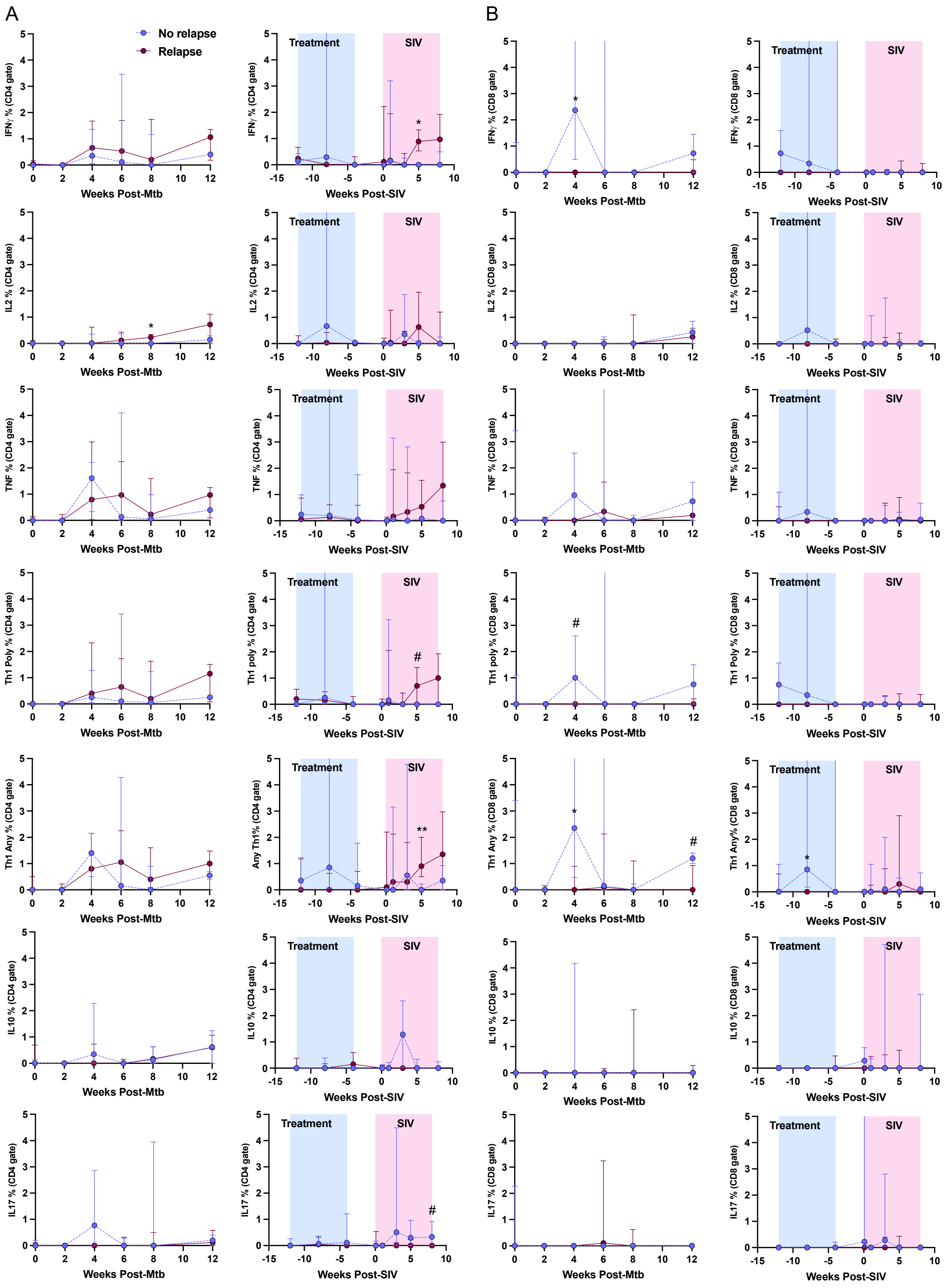

Supplementary Figure 17. Serial frequencies of Mtb-specific, central memory (CD45Ra-CD27+), Th1-, IL10-, and IL17-producing CD4 (A) and CD8 (B) T cells in the blood during Mtb infection, drug treatment, and SIV infection among non-relapse (n=4) and relapse (n=8) animals. Th1 Poly represents CD4 or CD8 T cells that produce two or more Th1 cytokines. Th1 Any represents CD4 or CD8 T cells that make at least one Th1 cytokine. Blue shaded area shows weeks of drug treatment, pink shaded area shows SIV-infection, medians shown with IQR. Mann-Whitney tests were run at each time point with no correction for multiple comparisons. $0.05 < p < 0.10$: #, $p < 0.05$: *.

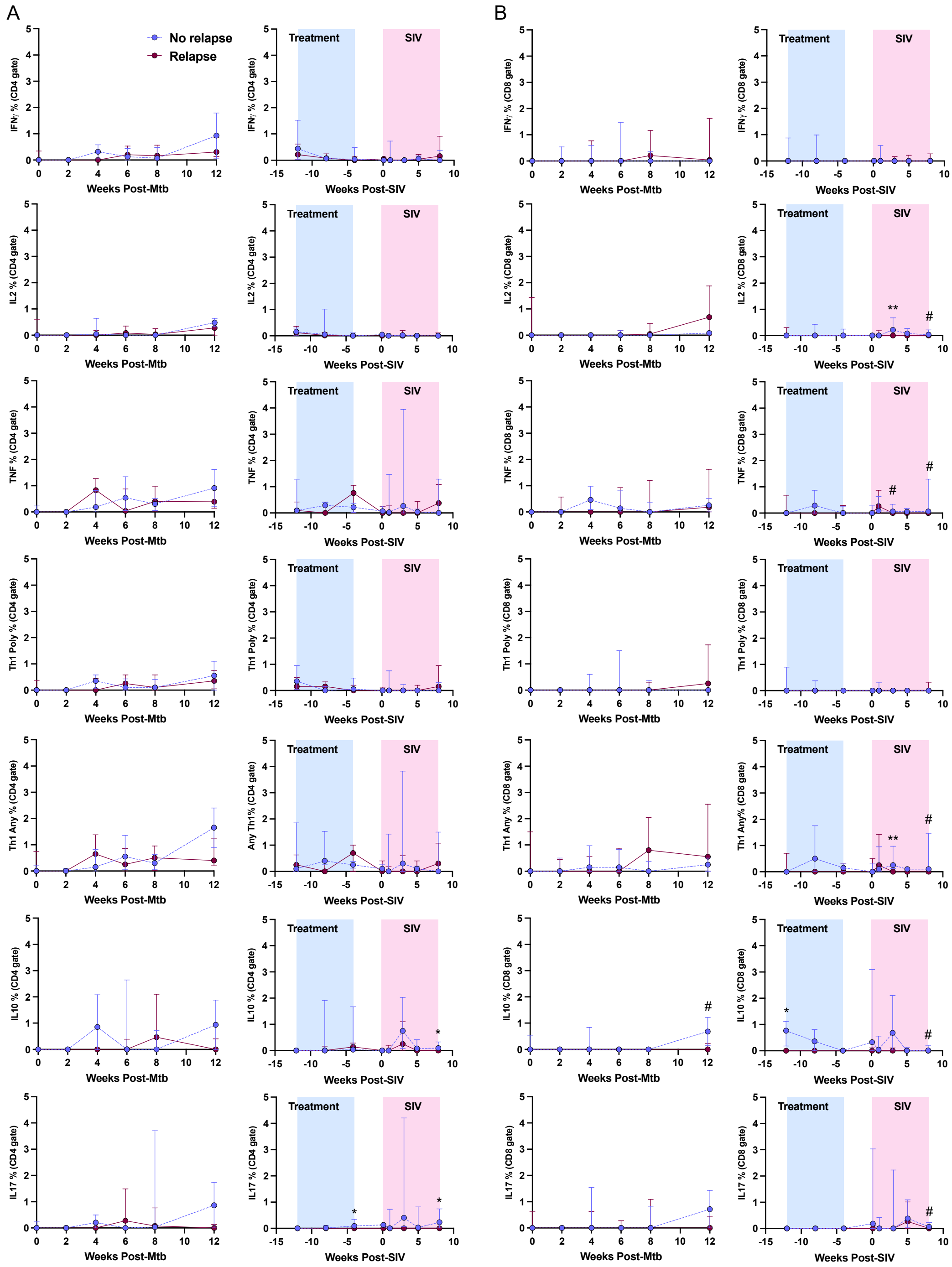

Supplementary Figure 18. Serial frequencies of Mtb-specific, effector memory (CD45Ra-CD27-), Th1-, IL10-, and IL17- producing CD4 (A) and CD8 (B) T cells in the blood during Mtb infection, drug treatment, and SIV infection among non-relapse (n=4) and relapse (n=8) animals. Th1 Poly represents CD4 or CD8 T cells that produce two or more Th1 cytokines. Th1 Any represents CD4 or CD8 T cells that make at least one Th1 cytokine. Blue shaded area shows weeks of drug treatment, pink shaded area shows SIV-infection, medians shown with IQR. Mann-Whitney tests were run at each time point with no correction for multiple comparisons. $0.05 < p < 0.10$: #, $p < 0.05$: *.

A. Effector Memory Cytolytic

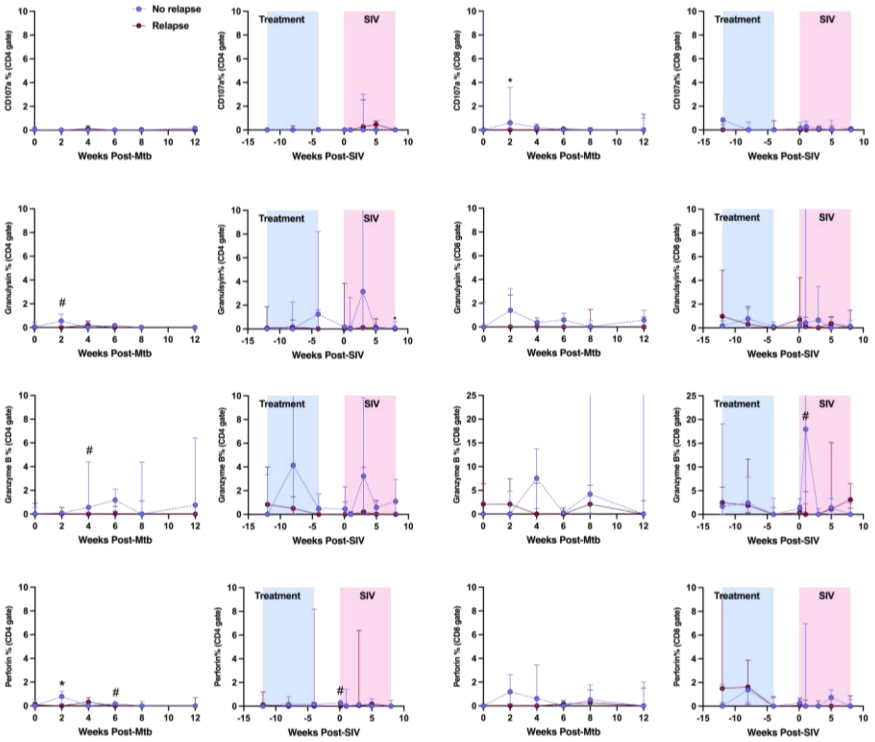

B. Terminal Differentiated Cytolytic

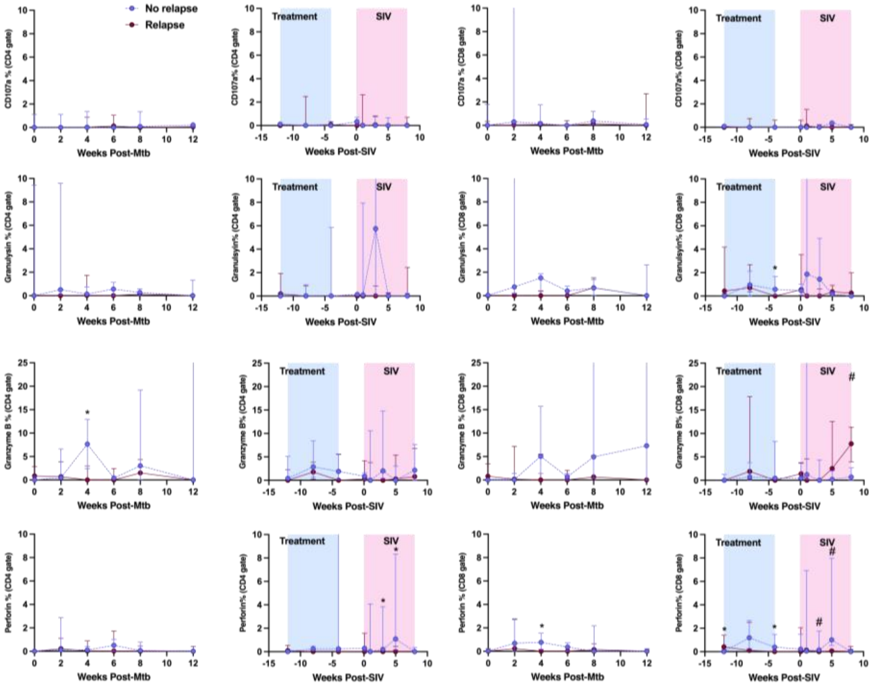

Supplementary Figure 19. (A) Serial frequencies of Mtb-specific, effector memory (CD45Ra-CD27-) CD4 and CD8 T cells with cytolytic characteristics in the blood during Mtb infection, drug treatment, and SIV infection among non-relapse (n=4) and relapse (n=8) animals. (B) Serial frequencies of Mtb-specific, terminal differentiated (CD45Ra+CD27-) CD4 and CD8 T cells with cytolytic characteristics in the blood during Mtb infection, drug treatment, and SIV infection among non-relapse (n=4) and relapse (n=8) animals. Blue shaded area shows weeks of drug treatment, pink shaded area shows SIV-infection, medians shown with IQR. Mann-Whitney tests were run at each time point with no correction for multiple comparisons. $0.05 < p < 0.10$: #, $p < 0.05$: *.

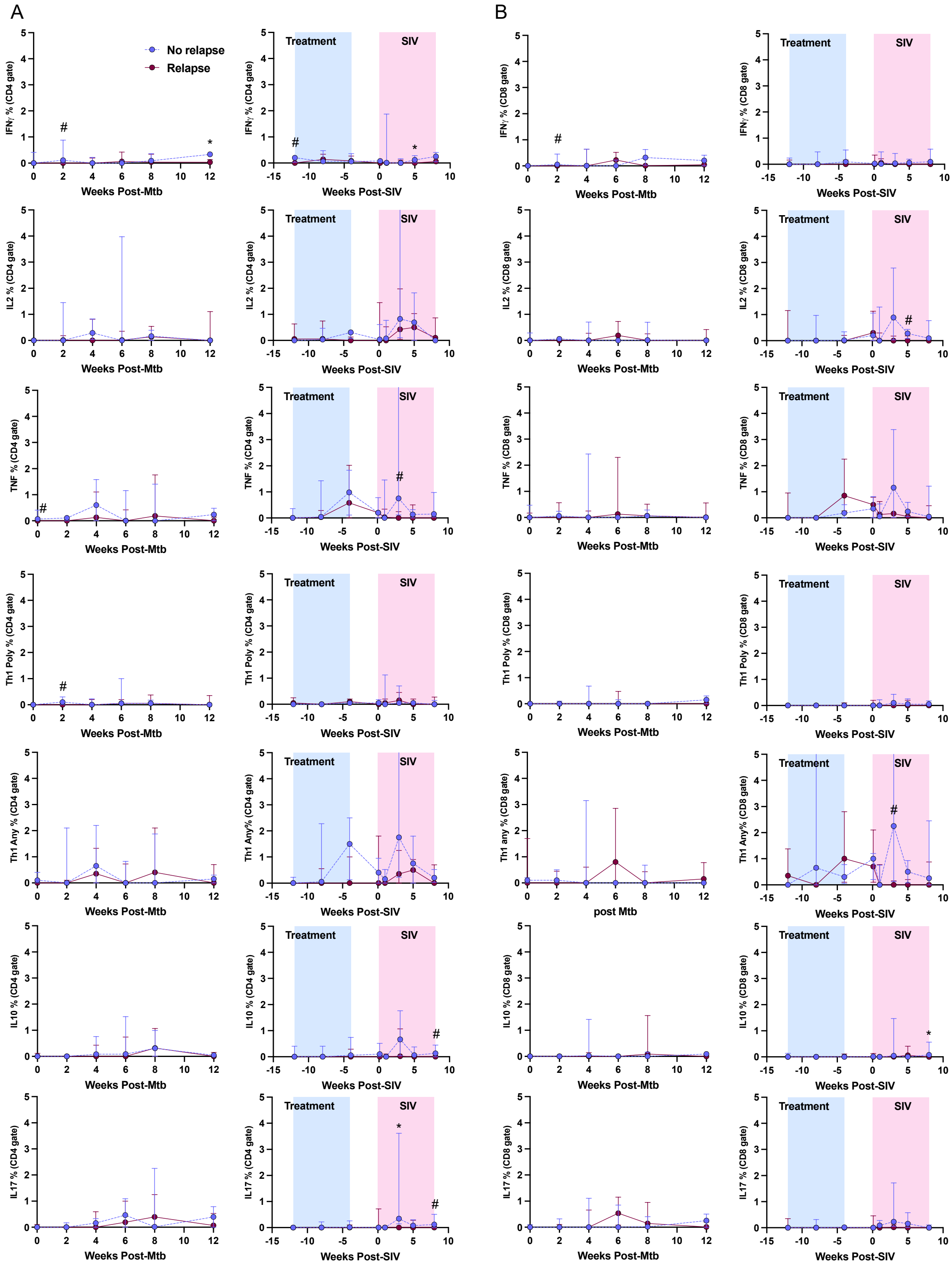

Supplementary Figure 20. Serial frequencies of Mtb-specific, terminal differentiated (CD45Ra+CD27-), Th1-, IL10-, and IL17-producing CD4 (A) and CD8 (B) T cells in the blood during Mtb infection, drug treatment, and SIV infection among non-relapse (n=4) and relapse (n=8) animals. Th1 Poly represents CD4 or CD8 T cells that produce two or more Th1 cytokines. Th1 Any represents CD4 or CD8 T cells that make at least one Th1 cytokine. Blue shaded area shows weeks of drug treatment, pink shaded area shows SIV-infection, medians shown with IQR. Mann-Whitney tests were run at each time point with no correction for multiple comparisons. $0.05 < p < 0.10$: #, $p < 0.05$: *.
